# The Draft Genome Sequence of the *Yersinia entomophaga* Entomopathogenic Type Strain MH96T

**DOI:** 10.3390/toxins8050143

**Published:** 2016-05-11

**Authors:** Mark R. H. Hurst, Amy Beattie, Eric Altermann, Roger M. Moraga, Lincoln A. Harper, Joanne Calder, Aurelie Laugraud

**Affiliations:** 1 AgResearch, Farm Systems & Environment, Lincoln Research Centre, Christchurch 8140, New Zealand; amy.beattie@agresearch.co.nz (A.B.); elphonso66@hotmail.com (L.A.H.); j.calder@middleton.school.nz (J.C.); 2 AgResearch Limited, Rumen Microbiology, Palmerston North 4474, New Zealand; eric.altermann@agresearch.co.nz; 3 Riddet Institute, Massey University, Palmerston North 4474, New Zealand; 4 AgResearch Limited, Bioinformatics & Statistics, Hamilton 3214, New Zealand; roger.moraga@agresearch.co.nz; 5 AgResearch Limited, Bioinformatics & Statistics, Lincoln Research Centre, Christchurch 8140, New Zealand; aurelie.laugraud@agresearch.co.nz

**Keywords:** *Yersinia entomophaga*, *Yersinia ruckeri*, *Yersinia nurmii*, Rhs, genome sequence, entomopathogen

## Abstract

Here we report the draft genome of *Yersinia entomophaga* type strain MH96T. The genome shows 93.8% nucleotide sequence identity to that of *Yersinia nurmii* type strain APN3a-cT, and comprises a single chromosome of approximately 4,275,531 bp. *In silico* analysis identified that, in addition to the previously documented *Y. entomophaga* Yen-TC gene cluster, the genome encodes a diverse array of toxins, including two type III secretion systems, and five rhs-associated gene clusters. As well as these multicomponent systems, several orthologs of known insect toxins, such as VIP2 toxin and the binary toxin PirAB, and distant orthologs of some mammalian toxins, including repeats-in-toxin, a cytolethal distending toxin, hemolysin-like genes and an adenylate cyclase were identified. The genome also contains a large number of hypothetical proteins and orthologs of known effector proteins, such as LopT, as well as genes encoding a wide range of proteolytic determinants, including metalloproteases and pathogen fitness determinants, such as genes involved in iron metabolism. The bioinformatic data derived from the current *in silico* analysis, along with previous information on the pathobiology of *Y. entomophaga* against its insect hosts, suggests that a number of these virulence systems are required for survival in the hemocoel and incapacitation of the insect host.

## 1. Introduction

*Yersinia* is a genus of Gram-negative facultative anaerobes belonging to the family Enterobacteriaceae, of the gamma subdivision of Proteobacteria [1]. The Yersiniae have undergone extensive diversification during the course of their evolution, with pathogenic *Yersinia* species such as *Y. pestis*, the causative agent of bubonic plague [2], and *Y. ruckeri*, which causes enteric redmouth disease in salmonid fish [3], along with nonpathogenic species, such as *Y. aldovae* [4], being identified. Recent phylogenetic analysis has shown that *Y. entomophaga* MH96^T^ is isogenic to *Y. nurmii* type strain APN3a-c^T^ [5], which was isolated in Finland from packages of broiler meat cuts packaged under a modified atmosphere [6]. These strains, along with *Y. ruckeri*, form a discrete clade away from other *Yersinia* species [7,8]. The placing of *Y. ruckeri* within the genus *Yersinia* is still a matter of conjecture [9,10]. 

*Y. entomophaga* was originally isolated from the cadaver of a New Zealand grass grub, *Costelytra zealandica* (Coleoptera: Scarabaeidae). *Y. entomophaga* is consistently pathogenic by *per os* challenge to this host, as well as a wide range of lepidopteran, coleopteran, and orthopteran species, with death of the insect typically occurring within 2–5 days of ingestion [11,12]*.* Aside from hemocytes, there are no obvious sites of *Y. entomophaga* colonization in the insect [12,13]. At 37 °C, aggregates of *Y. entomophaga* were observed both in the greater wax moth *Galleria mellonella* (Lepidoptera: Pyralidae) larval hemocoel and *in vitro* in Luria-Bertani broth. Electron microscopy of these *in vitro* cell aggregates revealed the presence of polar fimbriae [13].

The main virulence determinant of *Y. entomophaga* is an insect-active toxin complex (TC) derivative termed the Yen-TC, the genes encoding which are located on a pathogenicity island designated PAI_Ye96_ [14]. The Yen-TC is comprised of seven subunit proteins: two TC-A-like proteins (YenA1 and YenA2) and two chitinases (Chi1 and Chi2), which combine to form a pentameric cage into which the TC-B-like protein (YenB) and one of the two Rhs elements (TC-C-like proteins YenC1 and YenC2) bind to form the insect-active Yen-TC [15]. Rhs proteins are composed of a conserved *N*-terminal region and a divergent *C*-terminal “tip” region of 100–200 amino acids [16] that can encode the toxin effector component [17,18,19]. The Yen-TC causes loss of gut epithelial integrity, allowing the bacterium to gain entry to the insect hemocoelic cavity [13,20]. 

Once ingested, *Y. entomophaga* is likely to be confronted by a wide range of proteolytic gut enzymes, the endogenous microbial community, and physiological variables such as pH, ionic strength, and redox potential of the gut [21]. The epithelial and hemocoelic hemocytes of the insect gut produce large amounts of anti-microbial compounds and reactive oxygen species (ROS), which incapacitate invading pathogens [21,22]. Iron is a key element required by microbes and higher organisms. It is highly insoluble at a neutral pH, making the near neutral environment of the insect hemocoelic fluid [23] unsuitable for microorganisms [24,25]. In iron-limiting conditions, many pathogens produce siderophores, which are used to capture and sequester iron from the environment and eukaryotic intracellular protein iron storage molecules such as ferritin and transferrin [24]. 

The 4-day intra-hemocoelically-injected median lethal dose of either *Y. entomophaga* or its Yen-TC deletion derivative *Y. entomophaga* ΔTC towards *G. mellonella* is approximately three cells [13]. This low dose is similar to the number of *Photorhabdus* cells required to cause lethality. Once released into the hemocoelic cavity, the nematode-vectored *Photorhabdus* and *Xenorhabdus* species produce a range of toxins active in the hemoceol and antimicrobial compounds [26,27]. These include several TC orthologs [28], called the *Photorhabdus* insect-related binary toxins (PirAB), which are active when injected into the hemocoelic cavity of *G. mellonella* [29,30], and the apoptotic *P. luminescens*-derived Mcf (makes caterpillars floppy) protein [31,32]. In addition to these insect-active toxins, *P. luminescens* and *Xenorhabdus nematophila* secrete hydroxystilbene and benzylideneacetone, respectively, which limit the growth of competing microorganisms and inhibit phenol oxidase produced by phagocytic hemocytes of the insect [27,33,34]. 

The low number of *Y. entomophaga* cells required to cause an effect post-hemocoelic injection leads us to suggest that, similar to *Photorhabdus* and *Xenorhabdus* species, *Y. entomophaga* encodes and produces a wide range of factors that suppress the host immune system, and possibly limit the growth of the endemic microbial community [13]. To help elucidate the possible underlying mechanisms, a draft *Y. entomophaga* genome was assembled, allowing the *in silico*-based identification of possible virulence determinants. 

## 2. Results

### 2.1. *Yersinia entomophaga* Genome Summary

**Figure 1:**
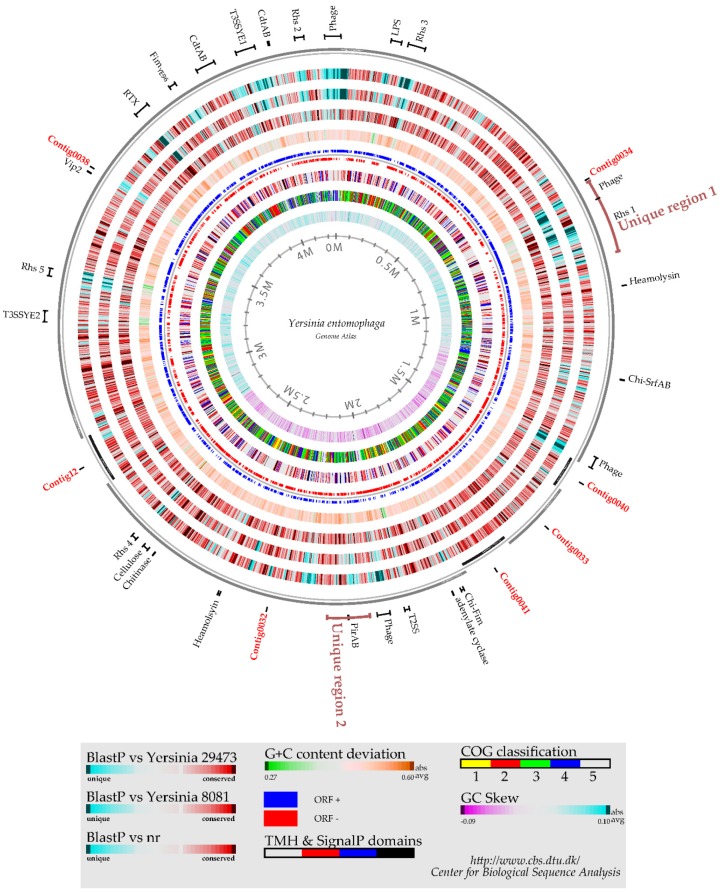
Genome Atlas diagram of the *Y. entomophaga* genome compared with those of *Y.* *enterocolitica* 8081 and *Y. ruckeri* ATCC 29473. The image represents a circular view of the complete genome sequence. **Innermost circle 1**: GC-Skew. **Circle 2**: COG classification: 1. information storage and processing; 2. cellular processes and signaling; 3. metabolism; 4. poorly characterized; and 5. ORFs with uncharacterized COGs or no COG assignment. **Circle 3**: Prediction of membrane-bound and cell surface proteins. White: no transmembrane helices (TMH) were identified. Black: ORFs with at least one TMH. Red: ORFs predicted to encompass a signal peptide sequence. Blue: ORFs predicted to incorporate both TMH and a signal peptide sequence. **Circle 4**: ORF orientation. ORFs in the sense orientation (ORF+) are shown in blue; ORFs oriented in the anti-sense direction (ORF−) are in red. **Circle 5**: G+C content deviation. Deviations from the average G+C content are shown in either green (low G+C spike) or orange (high G+C spike). **Circles 6–8**: BlastP comparison of the *Y. entomophaga* ORFeome with custom *Y. enterocolitica* 8081, *Y. ruckeri* ATCC 29473 Blast databases. Regions in blue represent unique proteins in *Y. entomophaga*, while highly conserved features are shown in red. The degree of color saturation corresponds with the level of similarity. **Circle 9**: Order of the numbered contigs comprising the draft genome. Contig names are shown in red, refer to Section 4.3 for contig information. Regions and ORFs of specific interest are shown at their respective genome location, bars are drawn to scale.

The draft *Yersinia entomophaga* genome sequence comprises one scaffold consisting of six contigs joined by PCR. The contig boundaries are indicated in Figure 1. Three gaps remain at approximately 1,413,485, 1,811,615, and 2,801,405 (gb|CP010029), and these DNA regions coincide with phage elements. 

The *Y. entomophaga* draft genome sequence comprises 4,275,531 bp, with a G+C content of 48.58%, with seven 16S rRNA ribosomal-associated regions. Analysis of the functional categories and genome-wide distribution of all unique genes with assigned clusters of orthologous groups (COGs) revealed a total of 4225 proteins. In total, 325 hypothetical proteins and 703 proteins not represented in the COG database, comprising 24.2% of the *Y. entomophaga* genome, were identified (Table 1). 

**Figure 2:**
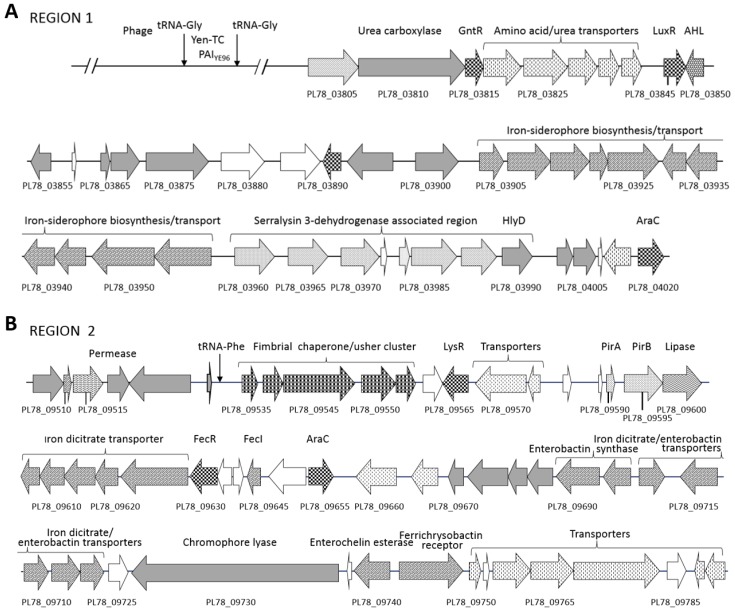
Schematic of virulence-associated Regions 1 (**A**) and 2 (**B**), which encode a range of toxin components, iron acquisition systems, and adhesin-encoding genes. The positions of Regions 1 and 2 relative to the draft *Y. entomophaga* genome sequence are shown in Figure 1. Arrows denote: speckled: putative virulence determinant; grey fill: characterized ortholog; horizontal waves: proteolytic/substrate binding protein; no fill: hypothetical protein; checkered: regulator protein; cross-hatched: fimbrial/pili-associated protein. Selected loci are labeled, refer to Table 1 for locus annotation.

*In silico* analysis revealed 93.8% DNA sequence identity between the *Y. entomophaga* genome and the draft genome sequence of *Y. nurmii* type strain APN3a-c^T^(gb|CIP110231). No region with significant DNA identity to the *Y. entomophaga* PAI_Ye96_ Yen-TC virulence-associated region was identified in the *Y. nurmii* APN3a-c^T^ genome sequence. Alignment of the *Y. entomophaga* ORFeome with the *Yersinia* species *Y. ruckeri* 29473 and *Y. enterocolitica* 8081 identified several genomic regions greater than 10 kb in size that were either unique or had an atypical G+C content compared with the rest of the *Y. entomophaga* genome sequence (Figure 1). Two regions of DNA, designated Region 1 and Region 2, were identified in the genome (Figure 2, Table S1). These regions encode a range of putative virulence factors and gene clusters involved in cell adhesin and in iron acquisition, which are outlined in detail below. 

### 2.2. Putative Virulence Clusters

Documented and putative *Y. entomophaga*-encoded toxins are listed in Table 2. 

### 2.3. Rhs and Type VI-Associated Regions 

**Figure 3:**
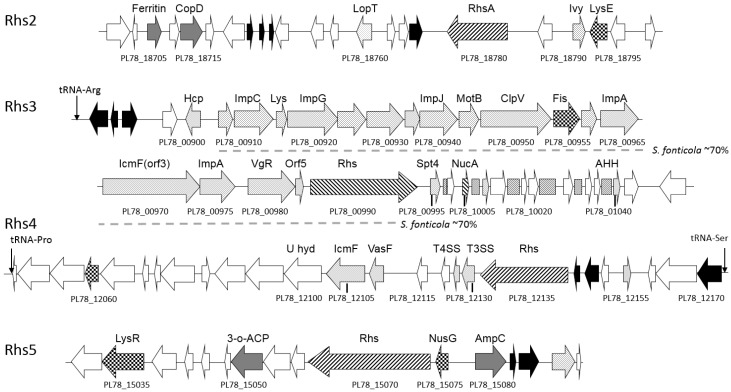
Schematic of *Y. entomophaga* Rhs-associated regions Rhs2–Rhs5. The positions of Rhs-associated regions relative to the draft *Y. entomophaga* genome sequence are shown in Figure 1. Arrows denote: speckled: putative virulence determinant; black fill: IS or transposon element; forward diagonal: Rhs element; grey fill: characterized ortholog; no fill: hypothetical protein; checkered: regulator. Dashed horizontal line denotes region of ~70% DNA identity of the Rhs-associated Region 3 to the *S. fonticola* strain DSM 4576 genome sequence (gb|CP011254.1)*.* Box foward diagonals located within Rhs-associated Region 3, denotes BlastX non functional translated Rhs similarty. tRNA loci are indicated, selected loci are labeled, refer to Table S2 for locus annotation.

Including the YenC1 and YenC2 subcomponents of the Yen-TC (Rhs-associated Region 1), the *Y. entomophaga* genome harbors five gene clusters encoding a Rhs element (Figure 3). Encoded within Rhs-associated Region 2 is a third YenC ortholog (PL78_18780; Figure 3), which shows high *N*-terminal amino acid similarity to each of the Yen-TC subcomponents (YenC1 and YenC2), and to TccC3 from Vibrio parahemolyticus, where the putative *C-*terminus effector is similar (Table S2). Located 5′ of PL78_18780 is PL78_18790, an ortholog of the *Escherichia coli* inhibitor of vertebrate lysozyme protein (Ivy) [35], a lysozyme that targets the peptidoglycan moiety of bacterial cell walls [36]. A region of DNA flanked by IS elements was identified 3′ of the Rhs element (PL78_18780). The DNA internal to these IS elements encodes several hypothetical proteins and a *Y. entomophaga* ortholog (PL78_18760) of the *Yersinia pekkanenii* and *P. luminescens* LopT type III secretion system (T3SS) effector proteins (Figure 3; Table S2).

Rhs-associated Region 3 contains a large gene cluster comprising loci with similarity to several type VI secretion system (T6SS) inner membrane protein (Imp) components, IcmF (PL78_00970), and a VgR locus (PL78_00980) (Table S2; Figure 3). A subset of these genes within Rhs-associated Region 3 share a similar gene order to the *P. luminescens pmt* genomic island, which encodes an *E. coli* macrophage toxin-like (mt-like) protein [37]. Though no mt-like protein was identified in the *Y. entomophaga* genome, several similarly oriented loci, some with partial homology to the Rhs core domain were identified 3ʹ of the Rhs element (PL78_00990) (Table S2). The *N* terminus of PL78_01005 showed partial identity to the Rhs core domain region, while its *C* terminus shares identity with the nuclease domain of the HNH/EndoVII superfamily (Table S2). Another locus (PL78_00995) showed amino acid similarity to the Spt4 eukaryotic transcription elongation factor domain (Figure 3; Table S2). BlastX analysis identified the regions in between loci (PL78_00995-PL78_01045) located 3′ of the Rhs element (PL78_00990) had non-functional translated similarity to the RhsA core region (Figure 3). Of interest, an 18,893-bp region (176,282–192,175 bp, *Y. entomophaga* genome sequence gb|CP010029), located within Rhs-associated Region 3, shares approximately 70%–72% DNA identity with the *S. fonticola* strain DSM 4576 genome sequence (gb|CP011254.1). 

Rhs-associated Region 4 encodes an ortholog (PL78_12135) of the *Yersinia pseudotuberculosis* RhsA wapA_1 protein (Table S2). Downstream of this ortholog is PL78_12130, which shares amino acid similarity with the T3SS effector protein from *Xanthomonas campestris* pv. *musacearum* NCPPB 4379 [38]. Similar to Rhs-associated Region 3, Rhs-associated Region 4 encodes an ortholog of the IcmF-T6SS-associated protein (PL78_12105) (Figure 3; Table S2). 

Rhs-associated Region 5 encodes the largest Rhs element (PL78_15070), consisting of 1,584 amino acids with high similarity to type IV secretion system protein RhsA from *Pseudomonas fluorescens* (Table S2). Several loci with protein orthologs in *Chromobacterium violaceum* and *P. luminescens* were identified downstream of PL78_15070 (Table S2). A NusG-type regulator with amino acid similarity to the *Serratia entomophila* *amb2* locus [39] was identified upstream of Rhs PL78_15070 (Table S2). However, *in silico* analysis did not identify a region corresponding to the operon polarity suppressor (5′-GGCGGTAGNNT-3′), to which the NusG regulator binds, within 15 kb of *Y. entomophaga* PL78_15075. Of note, the G+C content of Rhs Region 5 (G+C, 41.1%) was significantly divergent to that of the *Y. entomophaga* genome as a whole (G+C, 48.6%) (Figure 1), and had no significant nucleotide identity to DNA sequences deposited in the databases.

### 2.4. Type II and III Secretion Systems 

**Figure 4:**
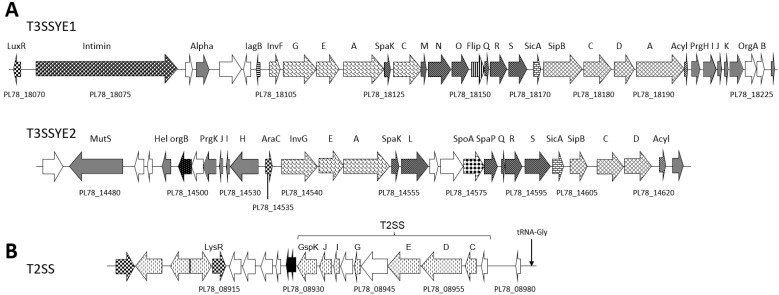
Schematic of the *Y. entomophaga* type II (T2SS) and III (T3SS) secretion systems. (**A**) the *Y. entomophaga* T3SSs T3SSYE1 and T3SSYE2. Orthologs shared between T3SSYE1 and T3SSYE2 are similarly shaded. (**B**) *Y. entomophaga* type II (T2SS). Arrows denote: black fill: IS or transposon element; grey fill: characterized ortholog; no fill: hypothetical protein; checkered: regulator protein; verticle dashes: cellular transport protein. tRNA loci are indicated, selected loci are labeled, refer to Table S3, for locus annotation. The positions of these protein secretion regions relative to the draft *Y. entomophaga* genome sequence are shown in Figure 1.

T3SS complexes enable bacteria to dock directly with host cells and actively deliver toxin effectors into the target cell [40,41]. The *Y. entomophaga* genome encodes two T3SSs, designated T3SSYE1 and T3SSYE2 (Table S3, Figure 4A). The gene components of T3SSYE1 have significant amino acid similarity to the components of the *S. fonticola* strain DSM 4576 T3SS (Table S3), while the T3SSYE2 proteins showed the greatest similarity to T3SS orthologs of *Y. ruckeri* (Table S3). Based on amino acid similarity and relative ORF positions in comparison with other published T3SSs, T3SSYE1 and T3SSYE2 resemble the Inv-Mxi-Spa gene order of *Salmonella typhimurium* SPI-1 [42] and the gene order of the *Y. enterocolitica* 8081 and A127/90 *Yersinia* secretion apparatus (Ysa) present in biotype IB strains of *Y. enterocolitica* [42], respectively. PL78_18075, encoding an intestinal cellular attachment intimin protein [43], was located upstream of T3SSYE1 and in the same orientation. Similar to the *Y. enterocolitica* Ysa T3SS [42,44], the orientation of the ORFs encoding T3SSYE2 are divergent. The AraC-type regulator gene and the genes encoding InvG (PL78_14540) and the acyl carrier (PL78_14620) are similarly oriented, while the genes upstream of AraC (PL78_14535), in the region from *prgH* (PL78_14530) to *orgB* (PL78_14505), are oriented in the opposing direction (Figure 4A). Both T3SSYE1 and T3SSYE2 encode the invasin InvA, which, in other T3SSs, promotes transit of the pathogen across the host cell [41,43]. *In silico* analysis did not identify any loci with amino acid similarity to documented toxin orthologs within or in close proximity of either T3SSYE1 or T3SSYE2 (Figure 4A). 

The type II secretion system (T2SS) is an alternate delivery system enabling the transport of folded macromolecules such as toxins. T2SS’s comprise a composite of proteins that span both the inner and outer bacterial cell membranes [45,46]. We identified a single T2SS gene cluster in *Y. entomophaga*, which comprised 11 loci (PL78_08930–PL78_08980) with similarity to the T2SS of several related species, including *Y. ruckeri* (Figure 4B; Table S3). We noted that the DNA encoding the T2SS is of an atypically low G+C content (35%).

### 2.5. Accessory Virulence Determinants

*In silico* analysis of the *Y. entomophaga* sequence identified the presence of several other virulence determinants with orthologs in both insect and mammalian pathogens. The *Y. entomophaga* loci PL78_09590 and PL78_09595 share high amino acid similarity with the *P. luminescens* PirA and PirB binary toxins [29], respectively (Figure 2B; Table S1).

**Figure 5:**
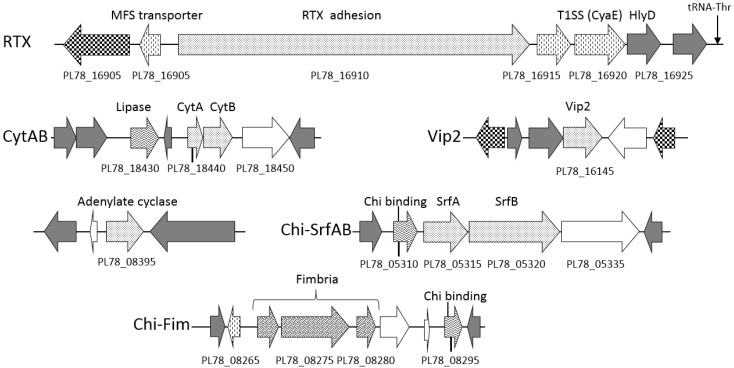
Schematic of putative *Y. entomophaga* accessory virulence determinants. The positions of these virulence determinants relative to the draft *Y. entomophaga* genome sequence are shown in Figure 1. Arrows denote: speckled: putative virulence determinant; grey fill: characterized ortholog; no fill: hypothetical protein; checkered: regulator protein; cross-hatched: fimbrial/pili-associated protein; horizontal waves: proteolytic/substrate binding protein; verticle dashes: cellular transport protein. Selected loci are labeled, refer to Table S4 for locus annotation.

The largest *Y. entomophaga* locus, PL78_16910, encodes a 4660-amino acid repeats-in-toxin (RTX) protein (Figure 5). Similar to other *rtx* gene clusters, which are relatively conserved at the amino acid level and in regards to gene order [47], loci involved in RTX transport were located both 5′ and 3′ of PL78_16910 (Figure 5; Table S4). 

The *Y. entomophaga* loci PL78_18440 and PL78_18445 are orthologs of CdtA and CdtB, respectively, which are subcomponents of the cytolethal distending toxin (Cdt) (Table S4). Typically, Cdts are encoded by three co-located genes, *cdtA*, *cdtB*, and *cdtC*, the CdtB component of which is the active toxin [46,47]. Interestingly, no CdtC ortholog was identified in *Y. entomophaga*. Although all mammalian Cdt orthologs contain an *N*-terminal signal peptide [48,49], only the *Y. entomophaga* CdtB contains a *N*-terminal signal peptide.

The *C*-terminal region of PL78_16145 showed partial amino acid similarity to the ADP ribosyltransferase domain of the VIP2 superfamily (Table S4) derived from the *Bacillus cereus* vegetative insecticidal protein VIP2 [50,51]. The binary VIP toxin is typically composed of two toxins, VIP1 and VIP2, and the VIP1 multimer enables the delivery of the VIP2 ADP-ribosylase to the target cell cytoplasm [52]. No VIP1 ortholog was identified in the *Y. entomophaga* genome. 

PL78_08395 was also identified as a putative toxin. It showed amino acid identity to an adenylate cyclase domain, with orthologs in several different bacterial genera (Figure 5, Table S4). This included a distant ortholog with less than 35% amino acid identity over 197 amino acid residues to the domain region of *Bacillus anthracis* adenylate cyclase [53]; in this instance, the *N*- and *C*-terminal regions of the non-domain regions were dissimilar (Table S4). We also note that, unlike *B. anthracis*, no corresponding protective antigen transport module [53] was identified in the *Y. entomophaga* genome.

### 2.6. Iron Acquisition 

Several iron-associated systems, including siderophores, heme-binding proteins, and their cognate receptors and regulators, were identified in the *Y. entomophaga* genome. Although heme- and iron-associated regions are distributed throughout the *Y. entomophaga* genome, these gene clusters are more prevalent in the vicinity of putative virulence components (Figure 2). We specifically noted loci involved in iron siderophore synthesis and transport within Region 1, which were located between the Yen-TC-encoding gene cluster and the later-outlined proteolytic serralysins (Figure 2A). Located within this region as well are the loci PL78_03845 and PL78_03850 (Figure 2A), which are orthologs of the *Y. ruckeri* population-dependent quorum sensing LuxR transcriptional regulator and the *N*-acyl homoserine lactone synthase, respectively (Table S1). We also identified genes involved in iron transport and enterobactin synthesis within Region 2, along with the cognate ferrichrysobactin receptor (PL78_09745). These genes were in proximity to *pirAB* (Figure 2B; Table 1). A single ferric uptake regulator (Fur) (PL78_01300), which represses specific genes in the presence of iron [24], was also identified in a non-descript region of the genome.

### 2.7. Proteolytic Enzymes

**Figure 6:**
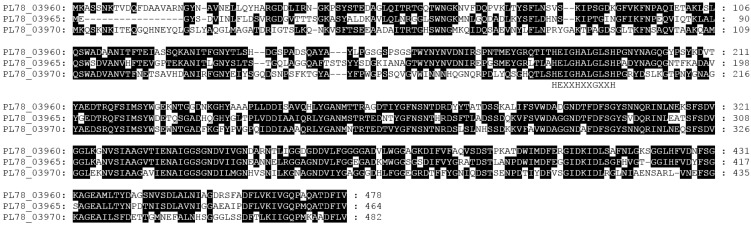
Amino acid sequence alignment of the *Y. entomophaga* serralysins PL78_03960; PL78_03965; PL78_03970. The Zn-endopeptidase metal-binding motif (HexxHxxGxxH) is indicated. Refer to Figure 2A for relative position of the serralysin-associated gene cluster within Region 1.

The production of pathogen-derived proteases, chitinases, esterases, and lipases can degrade microbial and host proteins and lyse host cells, liberating iron and other compounds. A *Y. entomophaga* operon containing three loci (PL78_03960, PL78_03965, PL78_03970), each encoding a serralysin metalloprotease with at least 68% amino acid sequence similarity to each other, was identified within Region 1 (Figure 2; Table S1). Amino acid alignment of the *Y. entomophaga* serralysin gene cluster with those from closely related species identified slight amino acid divergence across the entire amino acid sequence, although each contained the Zn-endopeptidase metal-binding motif (HexxHxxGxxH). The *Y. entomophaga* ORF PL78_03965 is truncated by 17 *N*-terminal amino acid residues (Figure 6). An alkaline protease inhibitor (PL78_03980) was located 3′ of the *Y. entomophaga* serralysin gene cluster (Figure 2A), while hemolysin transporter HlyD (PL78_03990) was located downstream of PL78_03980. In addition to degradative enzymes (Table S4), the *Y. entomophaga* genome encodes two hemolysin genes (PL78_04365, PL78_11060) with a high level of amino acid similarity to hemolysins from *Y. ruckeri* (Table S4). A thermolabile hemolysin (PL78_09600) with identity to a lipase/hydrolase domain, and PL78_18430, which also has amino acid identity to the esterase/lipase domain, were located 3′ of the *pirAB* orthologs and 5′ of CdtA, respectively (Figure 2B; Figure 5).

Aside from the previously documented Yen-TC Chi1 and Chi2 proteins [14,17], a third predicted chitinase, PL78_11910, was also identified (Figure 1; Table S4). Two *Y. entomophaga* chitin-binding loci (PL78_05310, PL78_08295) with orthologs in *Y. intermedia* and *Y. ruckeri*, respectively, were also identified. The first, PL78_05310, is positioned upstream of PL78_05315 and PL78_05320, the respective orthologs of which (*srfA* and *srfB*) are found in the *S.* *typhimurium* pathogenicity island SPI-2. Transcription of *srfA* and *srfB* is upregulated in epithelial and macrophage cells [54]. The loci PL78_05315 and PL78_05320 are positioned 5′ of an undefined virulence-associated locus (PL78_10835) (Figure 5; Table S4). The second chitin-binding locus, PL78_08295, was located downstream of components of a fimbrial pilus cluster comprising three loci PL78_08270, PL78_08275, and PL78_08280, (Figure 5; Table S4).

### 2.8. Cell Adhesins 

**Figure 7:**
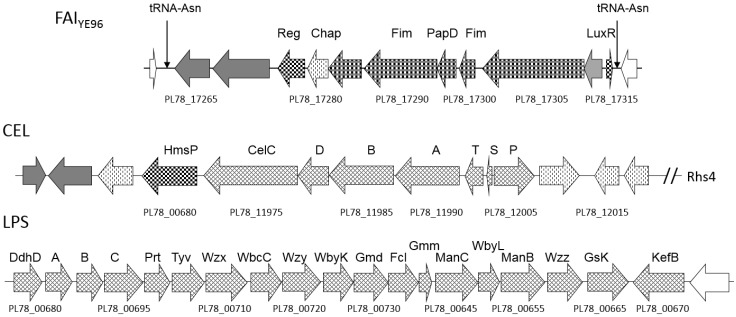
Schematic of selected *Y. entomophaga* cell surface appendage-encoding genes. Fimbrial-associated island (FAI_YE96_)-, cellulose gene cluster (CEL)-, and the lipopolysaccharide (LPS)-encoding regions. The positions of these regions relative to the draft *Y. entomophaga* genome sequence are shown in Figure 1. Arrows denote: checkered: regulator; cross-hatched: fimbrial/pili-associated protein; verticle dashes: cellular transport; grey fill: characterized ortholog; no fill: hypothetical protein; tRNA loci are indicated, selected loci are labeled, refer to Table S5 for locus annotation.

A prerequisite of virulence is a close association between the pathogen and the host cell. Some cell wall components, such as fimbriae or pili, form appendages that can aid in adherence to the host cell and enhance virulence. *In silico* analysis of the *Y. entomophaga* genome identified several fimbrial gene clusters and sub-component fimbrial gene clusters (Tables S1, S4 and S5). The most interesting of these comprised four fimbrial orthologs (PL78_17285-PL78_17300) that, along with chaperones and regulators, are predicted to be components of a putative fimbrial island cluster spanning 16,470 bp, flanked by two tRNA Asn 138-bp repeats (Figure 7, Table S5). Although the G+C composition (49.2%) of this region was similar to that of *Y. entomophaga* as a whole, the region shared the highest level of DNA identity (~72%) with *Y. intermedia* strain Y228 (gb|CP009801.1). This region was designated fimbrial-associated island FAI_YE96_. Similar to iron clusters, many of these fimbrial gene clusters are co-located with virulence-associated genes (Figure 2), with several fimbrial ushers and chaperones positioned upstream of the *pirAB* genes (Figure 2B; Table S1). 

Components of the lipopolysaccharide (LPS) gene cluster, PL78_00685-PL78_00780 (Figure 7), in the *Y. entomophaga* genome were most similar in amino acid identity and gene order to the *Y. ruckeri* LPS clusters (Table S5). Of note, the cellulose-encoding gene cluster, also with high amino acid identity to *Y. ruckeri*, was located within 15-kb of Rhs-associated Region 4, co-located with a large number of peptide transport proteins (Figure 1 and Figure 7; Table S2). 

### 2.9. Host Defense and Microbial Competition Systems

The *Y. entomophaga* genome encodes several loci that are likely to assist in the survival of the bacterium in the insect host. The genome encodes a colicin V protein (PL78_16525), two non-ribosomal peptide synthases (PL78_04165 and PL78_15615) that are likely to direct the production of microbial- and or host-active metabolites (Table S6). A second Ivy locus (PL78_15610) is located 5′ of PL78_15615. A number of ROS defense loci were also identified (Table S6), including superoxide dismutases (Sod) loci: an iron binding SodB (PL78_05910), a manganese-binding SodA (PL78_12235), and a copper/zinc binding Sod (PL78_14360). Each of these proteins has the capacity to catalyze the conversion of superoxide radicals to molecular oxygen.

## 3. Discussion 

Bioinformatic analysis of the draft *Y. entomophaga* genome sequence identified a wide range of toxin-encoding regions, as well as gene clusters involved in iron capture and transport. Although the assessed ORFeome identity indicates that *Y. entomophaga* is more closely related to *Y. ruckeri* and *Y. enterocolitica* (Tables S1–S6), the main virulence determinants of these species, such as the Afp18 [55] and YadA or Yop effector orthologs [56,57], were not identified in the *Y. entomophaga* draft genome sequence. With respect to known entomopathogenic toxins, *in silico* analysis of the *Y. entomophaga* genome did not identify orthologs of the *Pseudomonas entomophila* pore-forming toxin monolysin [58], the *P. luminescens* Mcf [31], or Afp/PVC proteins [59], the *X. nematophila* XaxA and XaxB binary toxins [60], or the *Xenorhabdus* Txp40 toxin [61]. Nor were any *Bacillus thuringiensis* toxin [50] orthologs identified. In contrast to *P. luminescens* and *Xenorhabdus* species, the *Y. entomophaga* genome encodes a single TC cluster, which may reflect the broad host range of the Yen-TC [14]. 

Of note, the *Y. entomophaga* genome encodes orthologs of the *Photorhabdus* hemocoelically-active PirAB binary toxin [30], based on the high amino acid sequence similarity, the *Y. entomophaga* PirAB orthologues are also likely to be hemocoelically active. An RTX-family ortholog was also identified in *Y. entomophaga*, members of which typically disrupt the host cell membrane, enhancing the capacity of the pathogen to colonize the host [47]. Based on the conserved function of RTX proteins, we predict a similar function for the *Y. entomophaga* RTX protein. 

### 3.1. Multi-Component Toxin Delivery Systems

The *Y. entomophaga* genome encodes several multi-component toxin delivery systems. Unlike *P. luminescens*, which contains a single T3SS (29), *Y. entomophaga* encodes two T3SSs. *Y. entomophaga* T3SSYE1 has a gene order similar to that of *S.* *typhimurium* T3SS SPI1, which is required for entry into intestinal epithelial cells. Once internalized, the effectors from the second *S.* *typhimurium* T3SS, SPI2, are released. This leads to the formation of a specialized *Salmonella*-containing vacuole where the bacterium can reside and replicate [62]. We note that the *Y. entomophaga* T3SSYE1 is associated with an intimin protein, which in other systems facilitates the attachment of the pathogen to the intestinal epithelial cells [43]. In this respect, we note that the closest *Y. entomophaga* initimin BlastP orthologs were in non-*Yersinia* species, including insect-associated bacteria such as *Xenorhabdus bovienii* and *Sodalis praecaptivus* (Table S3). The *Y*. *enterocolitica* Ysa system (T3SS), which has a gene order resembling that of T3SSYE2, is implicated in the initial colonization of the animal intestinal ileum [63] and has been found essential for the intracellular replication of *Y*. *enterocolitica* in *Drosophila melanogaster* S2 cells, with the authors suggesting that *Y*. *enterocolitica* has an insect host [44]. In instances where T3SSs have been identified in entomopathogenic bacteria, including *P. luminescens* and *Pseudomonas aeruginosa*, they are active against hemocytes [64]. In this context, we note that *Y. entomophaga* has previously been observed within insect hemocytes [13]. 

In addition to the T3SSs, five gene clusters containing Rhs elements were identified, which, based on *in silico* analysis, are likely to play a significant role in virulence*.* The most prominent of these is Rhs-associated Region 3, which encodes components of a T6SS and several loci PL78_00995, PL78_01005, and PL78_01040 with similarity to different DNA-associating proteins, suggesting that these proteins are likely to interact with host cell DNA and a requirement for their delivery to a target cell. Although there are several prokaryotic orthologs in the current databases (Table S2), a protein with a Spt4 domain has not been previously documented in prokaryotic systems [65]. We surmise that, if internalized, PL78_00995 could affect eukaryotic cell viability through transcriptional activation. Of interest was the presence of DNA between each loci located 3′ of PL78_00990 that had translated similarity to the Rhs core domain region; a phenomenon that may suggest to the possible gene acquisition by PL78_00990. Rhs-associated Region 4 also encodes component orthologs of T6SS and a putative T3SS effector proteins. The region may therefore represent a *Y. entomophaga* variant of a T6SS (Figure 3). The two remaining Rhs-associated regions (2 and 5) encode Rhs elements as well as a LopT toxin ortholog (66) and a known regulator of virulence (Amb2) [39], respectively. Based on the presence of these proteins in other documented systems, these Rhs-associated regions are also likely to have a role in virulence in *Y. entomophaga*.

*In silico* analysis showed that many of the putative *Y. entomophaga* toxins, such as CdtB (PL78_18445), VIP2 (PL78_16145), Rhs (PL78_18780), LopT (PL78_18760), and the adenylate cyclase toxin (PL78_08395), are not accompanied by their associated transport module. It is plausible that some of these putative toxin effectors are co-opted by existing transport modules, such as the T3SSs or T6SS’s, enabling their delivery. The *P. luminescens* LopT effector, which in *Spodoptera littoralis* and *Locusta migratoria* is activated at sites of cellular defense to prevent phagocytosis, complements the *Y. pseudotuberculosis* T3SS, allowing its delivery into mammalian cells [66]. The Rhs TC-C-like component (PL78_18780) may also complement the YenC1 or YenC2 subcomponents. Alternatively, these putative *Y. entomophaga* toxins may be released directly into the eukaryotic cell cytosol by a yet to be determined mechanism. This could include the *Y. entomophaga* T2SS, whose role in other systems has yet to be fully elucidated, but has been implicated in the secretion of cell surface-located toxins and adhesins [45,67].

### 3.2. Co-Location of Cell Adhesins or Lytic Enzymes with Virulence Determinants 

*In silico* analysis identified several cellular attachment systems, including pili, LPS, fimbrial clusters, and components thereof, which are likely to form a vestigial adhesin appendage. Although we have previously noted polar fimbriae on bacterial aggregates grown at 37 °C [13], the function of these systems in regards to virulence has yet to be elucidated. *In silico* analysis revealed that many of the *Y. entomophaga* fimbrial genes and degradative enzymes, including chitinases, hemolysins, lipases, and proteases, are co-located with iron-associated siderophores and virulence-encoding gene clusters specifically in Regions 1 and 2 (Figure 2 and Figure 5). It has previously been noted that chitinases not only hydrolyze chitin, but also have binding affinity for mammalian and invertebrate LacdiNAc and LacNAc glycans, and are proposed to enhance binding of the pathogen to the host cell surface [68]. In addition to fimbriae, the proximity of some of these proteolytic enzymes to toxin clusters, specifically *pirAB* and *cdtAB*, with no detected transport system may be indicative of a secondary role, such as aiding the binding of the pathogen or toxin to a host cell. 

Host cell lysis by proteolytic enzymes or toxins results in the release of iron. We noted that three serralysin metalloprotease loci were co-located with a *Y. entomophaga* gene cluster involved in iron siderophore biosynthesis and transport in Region 2 (Figure 2B). Intra-hemocoelic injection of the *P. luminescens* serralysin (PrtS) causes melaninization of *G. mellonella* and *D. melanogaster* larvae [69]. A serralysin (Ser) from *Serratia marcescens* induces hemolymph bleeding in the silkworm *Bombyx mori*, possibly through the degradation of host-derived factors required for coagulation [70]. Massaoud *et al.* [71] determined that serralysins from different *Xenorhabdus* species with a high degree of amino acid similarity differ in their relative proteolytic efficiency in the presence of different chelation factors. This data would account for the presence of three co-located *Y. entomophaga* serralysin loci, each of which may be tailored to a specific host system. Based in the aforementioned scenarios the co-location of putative toxin, adhesion or lytic enzymes within Region 1 and Region 2 may reflect a subtle interplay between the proteins throughout the virulence process.

Several host defense systems identified in the current study, including Ivy, ROS defense systems, siderophores, and antimicrobial factors, have been documented in other systems [26,27]. Although the role of these components, along with a number of non-ribosomal peptide synthases, free radical sequestering systems, and LPS and cellulose gene clusters, has yet to be elucidated, orthologs in other systems [26,27,72] indicate that they are likely to play a significant role in survival of the bacterium post-ingestion or following entry into the insect hemocoelic cavity and/or the eukaryotic cell. 

In recent years, it has become apparent that many plant- and insect-active toxins have distant mammalian orthologs, with both plant and invertebrate pathogens proposed as the progenitors of mammalian pathogens [73,74,75,76,77]. Bioinformatic analysis of the *Y. entomophaga* genome did not identify any orthologs of known *Y. pestis* or *Y. pseudotuberculosis* virulence determinants*.* Those orthologs of known mammalian toxins that were identified, including hemolysin and adenylate cyclase toxin, had either low amino acid similarity or represented a sub-component of a composite toxin cluster. In this context, the ability of a toxin to traverse the mammalian mucosal intestinal lining or cross the chitinous insect intestinal tract relies on different delivery vehicles, which, when docked, enable the transfer of conserved effector toxin molecules that typically act to cause a similar subcellular effect [78,79]. In this respect, we note that specific effector molecules, such as the cytotoxic necrotizing factor 1 (Cnf1) domain of the Yen-TC YenC1 *C*-terminus, thought to be transported by the YenA and YenB components [17], are vectored by alternate transport modules, such as the *Photorhadus* virulence cassette [80]. However, *E. coli-*derived Cnf harbors its own amino terminal receptor-targeting domain [81].

*In silico* analysis of the *Y. entomophaga* draft genome sequence confirmed that the bacterium encodes a diverse range of putative effector toxins and proteolytic enzymes, which along with complex export machinery and iron-sequestering factors, reflects a finely tuned pathogen capable of incapacitating a wide range of insect species. The wide variety of toxins produced by *P. luminescens* and *Xenorhabdus* species, and encoded by *Y. entomophaga*, is likely to reflect the broad and non-discriminant nature of their associated nematode [26,82] and, in the case of *Y. entomophaga*, the broad host range of the Yen-TC. For these bacteria, the associated nematode or the Yen-TC mediate the entry of the pathogen into the hemocoelic cavity, from where infection can occur. The *Y. entomophaga* genome also encodes a large number of hypothetical proteins, the functions of which have yet to be defined. To gain greater insight into the full functional potential of the genome, a range of *in vitro* and *in vivo* transcriptomic and proteomic studies will need to be undertaken. 

## 4. Experimental Section

### 4.1. Genomic DNA Isolation 

Genomic DNA was prepared using a Bioline Isolate II Genomic DNA kit (BIO-52066), mini-spin column isolation. The yield and purity of the DNA was determined for 5-μL samples by electrophoresis on 0.8% agarose gel and by spectrophotometry on a NanoDrop 1000 apparatus (Thermo Scientific, Waltham, MA, USA). 

### 4.2. Genome Sequencing

Genome sequencing was undertaken at the University of Liverpool Centre for Genomic Research, Liverpool, United Kingdom. Eight kilobase paired-end libraries were generated using the Roche “Paired End Rapid Library Preparation Method Manual, 20 kb and 8 kb Span, April 2012” according to manufacturers instructions. Each library was quantified using Qubit and the size distribution assessed using the Agilent 2100 Bioanalyzer. The resultant libraries were further quantified by qPCR using a Roche LightCycler 480 and Kapa 454 library quantification kit. Medium volume emPCRs were performed for each library with 0.6 copies per bead of input material and recovery levels of beads confirmed using a Beckman Coulter Counter. Beads from emPCR reactions for each library were pooled and sequencing performed on half a picotiter plate according to manufacturers instructions using xlr70 (Titanium) chemistry. The genome sequencing gaps were closed in-house using PCR-based amplicon sequencing. PCR primer sets were manually designed or designed using Primer3 version 0.4.0 (Whitehead Institute for Biomedical Research, Cambridge, MA, USA) [83]. 

### 4.3. Genome Assembly 

For assembly, a combination of illumina single-end and Roche 454 pair-end data was used. Illumina data was trimmed for quality using SolexaQA version 2 software (Massey University, New Zealand, 2010), using DynamicTrim [84], with parameter *p* = 0.05, then normalized down to 30× coverage using the khmer digital normalization package [85], and the combined data was assembled using Newbler GS De Novo Assembler version 2.7 [86], run in homozygous mode, with overlap identity of 95%, an identity score of 1, and a difference score of −3. All contigs shorter than 1 kb were discarded as low coverage misassemblies after comparing them to the rest of the resulting assembly. This resulted in 6 paired-end contigs (Figure 1) as a single scaffold-containing 11 gaps. For gap closing, a collection of primers designed around the estimated gap positions and lengths was produced, and sequences were generated using Sanger sequencing. The scaffolded assembly, as well as Sanger reads, were imported into Geneious 7 (Biomatters Ltd., Auckland, New Zealand, 2013) [87], and assembled using the Medium Sensitivity/Fast parameters. The draft *Y. entomophaga* MH96 genome sequence is deposited in Genbank, as accession number: gb|CP010029; BioProject: PRJNA267025 and BioSample: SAMN03177402.

### 4.4. Genome Annotation

The genome was automatically annotated using the NCBI pipeline [88]. The COG categories were found using the standalone COG software [89]. Tables S1–S6 were manually curated.

### 4.5. Genome Atlas and Genome–Genome DNA Identity Comparison

The assembled draft genome was annotated using an updated version of the GAMOLA software suite [90] and results then processed for graphical presentation. The circle was created using GeneWiz browser 0.91 (http://www.cbs.dtu.dk/services/gwBrowser/) [91] and in-house developed software (version 2.3, AgResearch, Palmerston North, New Zealand, 2015). Circles 6–8, Blast similarities, were created using the NCBI non-redundant amino-acid Blast database, a custom *Y. enterocolitica* 8081, and a custom *Y. ruckeri* ATCC 29473 Blast database. Deduced amino acid sequences of the *Y. entomophaga* ORFeome were compared against the respective database using gapped BlastP (Version 2.2.24, NCBI, Bethesda, MD, USA, 2010) [92]. The draft *Y. entomophaga* MH96 genome sequence was compared by Latz (Version 1.02.00, Miller Lab, Penn state University, Philadelphia, PA, USA, 2010) [93] against the *Y. nurmii* type strain APN3a-c whole genome shotgun sequencing project (NZ_CPYD00000000).

## Figures and Tables

**Table 1:** Functional categories of *Yersinia entomophaga* clusters of orthologous groups (COGs).

Code	Value	% of Total	COG Category
A	2	0.05	RNA processing and modification
B	0	0.0	Chromatin structure and dynamics
C	197	4.7	Energy production and conversion
D	32	0.7	Cell cycle control, cell division, chromosome partitioning
E	324	7.6	Amino acid transport and metabolism
F	88	2.1	Nucleotide transport and metabolism
G	238	5.6	Carbohydrate transport and metabolism
H	157	3.7	Coenzyme transport and metabolism
I	91	2.2	Lipid transport and metabolism
J	180	4.2	Translation, ribosomal structure, and biogenesis
K	250	5.9	Transcription
L	161	3.8	Replication, recombination, and repair
M	208	4.9	Cell wall/membrane/envelope biogenesis
N	105	2.5	Cell motility
O	129	3	Posttranslational modification, protein turnover, chaperones
P	239	5.7	Inorganic ion transport and metabolism
Q	71	1.7	Secondary metabolite biosynthesis, transport, and catabolism
R	359	8.5	General function prediction only
S	325	7.7	Function unknown
T	193	4.5	Signal transduction mechanisms
U	130	3.1	Intracellular trafficking, secretion, and vesicular transport
V	43	1	Defense mechanisms
-	703	16.6	Not in COGs
Total	4225	-	-

**Table 2:** Known and putative *Y. entomophaga* toxin components.

Putative Toxin or Toxin Encoding Gene Cluster	Locus (Putative Virulence-Associated Region) ^1^	Predicted Function
Rhs1 (Yen-TC)	PL78_03740-03770	orally active toxin complex
Rhs2 (LopT)	PL78_18780 (PL78_18715-18790)	Hemoceolic active toxin
Rhs3 (Spt4)	PL78_00990 (PL78_00895-01045)	T6SS, hemoceolic active toxin
Rhs4 (T3SS, T6SS)	PL78_12135 (PL78_12045-12170)	Hemoceolic active toxin
Rhs5	PL78_15070 (PL78_15035-15075)	Effector island
YenT (Yst)	PL78_03785	Heat-stable enterotoxin
PirAB	PL78_09590-09595	Hemoceolic active toxin
CdtAB	PL78_18444-18445	Hemoceolic active toxin
RTX	PL78_16910	Repeats in toxin
adenylate cyclase	PL78_08395	Hemoceolic active toxin
Vip2	PL78_16145	Hemoceolic active toxin
LopT	PL78_18760	T3SS effector, hemoceolic active toxin
T3SS1	PL78_18075-18225	Type three secretion system
T3SS2	PL78_14485-14620	Type three secretion system

**^1^**Refer to Tables S1–S5 for locus annotation.

## Supplementary Materials

The following are available online at www.mdpi.com/2072-6651/8/5/143/s1, Table S1: *Yersinia entomophaga* unique Regions 1 and 2, Table S2: *Yersinia entomophaga* Rhs-associated regions 2–5, Table S3: *Yersinia entomophaga* Type 2 and 3-associated regions, Table S4: *Yersinia entomophaga* accessory virulence determinants: Repeats in Toxin cluster, CdtAB, VIP2, adenylate cyclase, chitinase, and chitin binding protein-associated regions and putative hemolysins, Table S5: *Yersinia entomophaga* cell adhesins, fimbriae, Lipopolysaccharide, and cellulose encoding clusters, Table S6: *Yersinia entomophaga* countermeasures: ColicinV, Sod, and non-ribosomal peptide synthetase.
