# Supplementary material for: The Draft Genome Sequence of the Yersinia entomophaga Entomopathogenic Type Strain MH96T
Source: Toxins (Basel). 2016 May 11;8(5):143. doi: 10.3390/toxins8050143 (PMC4885058; doi:10.3390/toxins8050143)
Supplement: Supplementary file 1 [file toxins-08-00143-s001.pdf]

Supplementary Materials: The Draft Genome Sequence of the *Yersinia entomophaga* Entomopathogenic Type Strain MH96T

Mark R. H. Hurst, Amy Beattie, Eric Altermann, Roger M. Moraga, Lincoln A. Harper, Joanne Calder and Aurelie Laugraud

Table S1. *Yersinia entomophaga* unique regions 1 and 2. For graphic depiction refer to Figure 2 of the associated manuscript.

Yersinia entomophaga unique region 1.

Locus (Amino Acid Residues)	Closest BlastP Orthologue [Species]<br>Length: Amino Acid Length of Orthologue<br>% Identity/% Similarity (Over the Indicated Range of Amino Acid Residues) in Relation to Target Sequence.<br>Protein Domain	Accession Number
PL78_03820 (423)	branched-chain amino acid ABC transporter substrate-binding protein [*Yersinia nurmii*]<br>Length: 423<br>95/97(423) 1–423	WP_049597723.1
	Aliphatic amidase expression-regulating protein [*Beauveria bassiana* D1-5]<br>Length: 648<br>92/95(420) 226–645	gbKGQ13304.1
PL78_03825 (524)	urea ABC transporter permease [*Yersinia nurmii*]<br>Length: 524<br>92/94(524) 1–524	WP_049597583.1
	MULTISPECIES: urea ABC transporter permease [*Enterobacteriaceae*]<br>Length: 524<br>73/81(507) 1–524	WP_048332627.1
PL78_03830 (357)	amino acid ABC transporter permease [*Yersinia nurmii*]<br>Length: 357<br>96/96(357) 1–357	WP_049597584.1
	amino acid ABC transporter permease [*Buttiauxella agrestis*]<br>Length: 357<br>88/93(357) 1–357	WP_034495296.1

PL78_03835 (265)	urea ABC transporter ATP-binding protein [*Yersinia nurmii*]<br>Length: 265<br>96/97(265)1–265	WP_049597585.1
	urea ABC transporter ATP-binding protein [*Cedecea neteri*]<br>Length: 265<br>89/94(265) 1–265	WP_038472477.1
PL78_03840 (232)	urea ABC transporter ATP-binding protein [*Yersinia nurmii*]<br>Length: 232<br>98/99(232) 1–232	WP_049597586.1
	urea ABC transporter ATP-binding protein [*Buttiauxella agrestis*]<br>Length: 232<br>86/93(232) 1–232	WP_034455625.1
PL78_03845 (261)	LuxR family transcriptional regulator [*Yersinia nurmii*]<br>Length: 247<br>98/99(247) 1–247	WP_049597724.1
	LuxR family transcriptional regulator [*Yersinia ruckeri*]<br>Length: 247<br>80/89(246) 1–247	WP_004719049.1
PL78_03850 (216)	acyl-homoserine-lactone synthase [*Yersinia nurmii*]<br>Length: 216<br>99/100(216)1–216	WP_049597587.1
	acyl-homoserine-lactone synthase [*Yersinia ruckeri*]<br>Length: 216<br>87/92(216) 1–216	WP_038245616.1
PL78_03855 (237)	membrane protein [*Yersinia nurmii*]<br>Length: 231<br>96/98(231) 1–231	WP_049597588.1
	hypothetical protein [*Yersinia intermedia*]<br>Length: 237<br>72/87(236) 1–236	WP_050077576.1

PL78_03860 (238)	hypothetical membrane protein [*Azoarcus* sp. BH72]<br>Length: 149<br>51/72(86) 68–149	embCAL95245.1
	hypothetical protein [*Photorhabdus luminescens*]<br>Length: 207<br>31/49(209)13–207	WP_052739685.1
PL78_03862 (81)	hydrolase [*Mesorhizobium loti*]<br>Length: 259<br>59/78(32)226–257	WP_027033575.1
	carbon monoxide dehydrogenase form II large subunit [uncultured bacterium]<br>Length: 315<br>37/53(52) 14–65	gbAAW66018.1
PL78_03865 (100)	ERF family protein [uncultured Mediterranean phage uvMED]<br>Length: 201<br>33/50(55) 41–94	dbjBAR33765.1
	PREDICTED: sarcoma antigen 1-like, partial [*Callithrix jacchus*]<br>Length: 276<br>33/54(46) 169–213	XP_008989763.1
PL78_03870 (336)	glycosyl transferase [*Yersinia nurmii*]<br>Length: 336<br>95/96(336) 1–336	WP_049597589.1
	glycosyl transferase [*Yersinia pseudotuberculosis*]<br>Length: 326<br>70/83(325) 1–336	WP_012303433.1
PL78_03875 (723)	dipeptidyl carboxypeptidase II [*Yersinia nurmii*]<br>Length: 723<br>95/97(723) 1–723	WP_049597590.1
	Dipeptidyl carboxypeptidase Dcp [*Yersinia ruckeri*]<br>Length: 729<br>82/89(726) 1–726	embCEK27267.1

PL78_03880 (519)	hypothetical protein [*Yersinia nurmii*]<br>Length: 519<br>97/97(519) 1–519	WP_049597591.1
	hypothetical protein [*Brenneria goodwinii*]<br>Length: 534<br>62/79(516)1–516	WP_048636529.1
PL78_03885 (453)	malate permease [*Yersinia nurmii*]<br>Length: 453<br>99/99(519) 1–453	WP_049597592.1
	malate permease [*Yersinia ruckeri*]<br>Length: 453<br>92/96(452) 1–452	WP_045844043.1
PL78_03890 (239)	transcriptional regulator [*Yersinia nurmii*]<br>Length: 239<br>99/99(239)1–239	WP_049597593.1
	transcriptional regulator [*Yersinia ruckeri*]<br>Length: 239<br>93/96(239) 1–239	WP_038242277.1
PL78_03895 (543)	histidine kinase [*Yersinia nurmii*]<br>Length: 543<br>97/98(543) 1–543	WP_049597594.1
	histidine kinase [*Yersinia ruckeri*]<br>Length: 543<br>87/94(543) 1–543	WP_042524781.1
PL78_03900 (470)	2-oxoglutarate translocator [*Yersinia nurmii*]<br>Length: 470<br>99/98(470) 1–470	WP_049597595.1
	2-oxoglutarate translocator [*Yersinia ruckeri*]<br>Length: 470<br>91/95(470)1–470	WP_038275783.1

PL78_03905 (254)	iron reductase [*Yersinia aldovae*]<br>Length: 254<br>94/96(254) 1–254	WP_042546885.1
	iron reductase [*Yersinia nurmii*]<br>Length: 254<br>94/98(254) 1–254	WP_049597596.1
PL78_03910(519)	cytochrome C biogenesis protein CcmH [*Yersinia aldovae*]<br>Length: 519<br>96/97(519) 1–519	WP_042546884.1
	cytochrome C biogenesis protein CcmH [*Yersinia nurmii*]<br>Length: 519<br>96/97(519) 1–519	WP_049597597.1
PL78_03915 (434)	putative siderophore biosynthetic enzyme [*Yersinia aldovae*]<br>Length: 434<br>97/98(434) 1–434	embCNL58692.1
	putative siderophore biosynthetic enzyme [*Yersinia nurmii*]<br>Length: 434<br>95/96(434) 1–434	embCNE39394.1
PL78_03920 (190)	siderophore biosynthesis protein [*Yersinia aldovae*]<br>Length: 198<br>95/97(197) 1–197	WP_049596217.1
	siderophore biosynthesis protein [*Yersinia aldovae*]<br>Length: 198<br>95/97(197) 1–197	WP_042546883.1
PL78_03925 (612)	siderophore biosynthesis protein IucA [*Yersinia aldovae*]<br>Length: 611<br>96/97(611) 1–611	WP_042839931.1
	siderophore biosynthesis protein IucA [*Yersinia aldovae*]<br>Length: 611<br>96/97(611) 1–611	WP_042546882.1

PL78_03930 (287)	putative iron-siderophore transport system %2CATP-binding component [*Yersinia aldovae*]<br>Length: 287<br>94/95(287) 1–287	embCNL53775.1
	putative iron-siderophore transport system %2CATP-binding component [*Yersinia aldovae*]<br>Length: 287<br>94/95(287) 1–287	embCNL58815.1
PL78_03935 (346)	iron ABC transporter permease [*Yersinia aldovae*]<br>Length: 346<br>97/97(216) 31–346	WP_049689070.1
	iron ABC transporter permease [*Yersinia nurmii*]<br>Length: 346<br>96/98(206) 41–346	WP_049597600.1
PL78_03940 (339)	iron ABC transporter permease [*Yersinia nurmii*]<br>Length: 339<br>94/95(339) 1–339	WP_049597601.1
	iron ABC transporter permease [*Yersinia aldovae*]<br>Length: 339<br>95/96(336) 1–339	WP_042839929.1
PL78_03945 (341)	periplasmic binding family protein [*Yersinia aldovae* 670-83]<br>Length: 37<br>96/97(376)1–376	gbAJJ64399.1
	iron-siderophore ABC transporter substrate-binding protein [*Yersinia aldovae*]<br>Length: 362<br>97/98(362)1–362	WP_042546879.1
PL78_03950 (749)	TonB-dependent receptor [*Yersinia aldovae*]<br>Length: 749<br>96/97(749) 1–749	WP_042839927.1
	TonB-dependent receptor [*Yersinia aldovae*]<br>Length: 749<br>96/97(749) 1–749	WP_042546878.1

PL78_03955 (609)	IucA/IucC family protein [*Yersinia aldovae*]<br>Length: 609<br>97/97(609) 1–609	WP_049596218.1
	siderophore biosynthesis protein IucA [*Yersinia aldovae*]<br>Length: 609<br>96/97(609) 1–609	WP_042546877.1
PL78_03960 (478)	serine 3-dehydrogenase [*Yersinia intermedia*]<br>Length: 479<br>86/93(478) 1–479	WP_050086263.1
	serine 3-dehydrogenase [*Yersinia nurmii*]<br>Length: 478<br>96/97(478)1–478	WP_049597605.1
PL78_03965 (464)	serine 3-dehydrogenase [*Yersinia nurmii*]<br>Length: 481<br>96/97(464) 18–481	WP_049597606.1
	serine 3-dehydrogenase [*Yersinia intermedia*]<br>Length: 485<br>80/87(464)1–485	WP_005182588.1
PL78_03970 (482)	metalloprotease [*Yersinia nurmii*]<br>Length: 482<br>93/95(482) 1–482	embCNE39855.1
	serine 3-dehydrogenase [*Yersinia ruckeri*]<br>Length: 477<br>55/67(442) 40–477	WP_042524782.1
PL78_03975 (77)	No similarity	-
PL78_03980 (109)	Proteinase inhibitor precursor [*Yersinia nurmii*]<br>Length: 138<br>97/100(109) 30–138	embCNE39896.1
	alkaline proteinase inhibitor [*Yersinia intermedia*]<br>Length: 138<br>78/87(109) 30–138	WP_050086261.1

PL78_03985 (580)	peptidase [*Yersinia nurmii*]<br>Length: 580<br>98/99(580) 1–580	WP_049597607.1
	peptidase [*Yersinia intermedia*]<br>Length: 580<br>91/94(580) 1–580	WP_050312322.1
PL78_03990 (434)	hemolysin D [*Yersinia nurmii*]<br>Length: 443<br>98/99(443) 1–443	WP_049597608.1
	hemolysin D [*Yersinia intermedia*]<br>Length: 443<br>87/93(443) 1–443<br>pfam00529	WP_050312323.1
PL78_03995 (440)	peptidase [*Yersinia nurmii*]<br>Length: 447<br>99/99(447) 1–447	WP_049597609.1
	peptidase [*Yersinia intermedia*]<br>Length: 447<br>91/96(447) 1–447	WP_050086258.1
PL78_04000 (195)	cytochrome B561 [*Yersinia nurmii*]<br>Length: 205<br>96/96 (2025) 1–205	WP_049597610.1
	cytochrome B561 [*Yersinia ruckeri*]<br>Length: 206<br>84/90(205) 2–206	WP_042524786.1

PL78_04005 (262)	hypothetical protein [*Yersinia nurmii*]<br>Length: 262<br>putative sulfite oxidase subunit YedY [*Yersinia nurmii*]<br>Sequence ID: emb|CNE40096.1| Length: 262<br>Number of Matches: 97/98(262) 1–262	WP_049597611.1
	hypothetical protein [*Yersinia ruckeri*]<br>Length: 262<br>90/94(262) 1–262	WP_042524787.1
PL78_04010 (112)	pentapeptide MXKDX repeat protein [*Yersinia nurmii*]<br>Length: 123<br>89/90(123) 1–123	embCNE40130.1
	hypothetical protein [*Yersinia ruckeri*]<br>Length: 112<br>77/90(112) 1–112	WP_042524788.1
PL78_04015 (300)	multidrug DMT transporter permease [*Yersinia nurmii*]<br>Length: 300<br>96/98(300) 1–300	WP_049597612.1
	membrane protein [*Yersinia ruckeri*]<br>Length: 300<br>87/93(289)	WP_004720598.1
PL78_04020 (292)	AraC family transcriptional regulator [*Yersinia nurmii*]<br>Length: 292<br>99/99(292) 1–292	WP_049597613.1
	AraC family transcriptional regulator [*Yersinia ruckeri*]<br>Length: 292<br>90/94(292) 1–292	WP_004720600.1

Yersinia entomophaga unique region 2

**Locus (amino acid resides)**	**Closest BlastP Orthologue [Species]<br>Length: Amino Acid Length of Orthologue<br>% identity/% Similarity (over the Indicated Range of Amino Acid Residues) in Relation to Target Sequence.<br>Protein Domain**	**Accession Number**
PL78_09500 (239)	tRNA (guanine-N7)-methyltransferase [*Yersinia nurmii*]<br>Length: 239<br>99/100(239) 1–239	WP_049599599.1
	tRNA (Guanine-N(7)-)-methyltransferase [*Yersinia ruckeri* ATCC 29473]<br>Length: 252<br>94/96(239) 14–252	gbEEP99613.1
PL78_09505 (366)	A/G-specific adenine glycosylase [*Yersinia nurmii*]<br>Length: 366<br>98/98(366) 1–366	WP_049599597.1
	A/G-specific adenine glycosylase [*Yersinia ruckeri*]<br>Length: 366<br>93/96(366) 1–366	WP_045844287.1
PL78_09510 (90)	oxidative damage protection protein [*Yersinia nurmii*]<br>Length: 90<br>99/98(90) 1–90	WP_049599595.1
	Fe(2+)-trafficking protein [*Yersinia massiliensis*]<br>Length: 90<br>97/98(90) 1–90	WP_019211664.1
PL78_09515 (358)	murein transglycosylase [*Yersinia nurmii*]<br>Length: 358<br>99/99(358) 1–358	WP_049599592.1
	murein transglycosylase [*Yersinia ruckeri*]<br>Length: 358<br>99/99(358) 1–358	WP_004719291.1

PL78_09520 (249)<br>Permease	permease [*Yersinia nurmii*]<br>Length: 249<br>94/95(249) 1–249	WP_049599589.1
	Sulfite exporter TauE/SafE [*Yersinia bercovieri*]<br>Length: 249<br>85/93(247) 1–247	embCNF01781.1
PL78_09525 (720)	ornithine decarboxylase [*Yersinia nurmii*]<br>Length: 720<br>97/98(719) 1–719	WP_049599586.1
	ornithine decarboxylase [*Yersinia pekkanenii*]<br>Length: 720<br>86/93(719) 1–719	WP_049615402.1
PL78_09550	tRNAphe	
PL78_09535 (176)<br>Fimbrial protein	fimbrial protein [*Yersinia nurmii*]<br>Length: 176<br>98/98(176) 1–176	WP_049597733.1
	fimbrial protein [*Yersinia intermedia*]<br>Length: 176<br>91/96(176) 1–176	WP_050076821.1
PL78_09540 (243)<br>Fimbrial chaperone	putative fimbrial chaperone [*Yersinia frederikseniir*]<br>Length: 243<br>89/95(243) 1–243	embCFR11699.1
	fimbrial chaperone protein [*Yersinia nurmii*]<br>Length: 242<br>98/97(242) 1–243	WP_049597734.1
PL78_09545 (887)<br>Fimbrial protein	fimbrial protein [*Yersinia nurmii*]<br>Length: 887<br>95/97(887) 1–887	WP_049597735.1
	fimbrial usher protein [*Yersinia frederikseniir*]<br>Length: 887<br>88/95(888) 1–887	WP_004710087.1

PL78_09550 (424)<br>Fimbrial usher protein StbD	putative fimbrial usher protein StbD [*Yersinia nurmii*]<br>Length: 455<br>96/98(455)1–455	embCNE45478.1
	fimbrial family protein [*Yersinia frederikseniir* ATCC 33641]<br>Length: 455<br>89/94(455) 1–455	gbKGA46118.1
PL78_09555 (243)<br>Chaperone	molecular chaperone [*Yersinia nurmii*]<br>Length: 255<br>95/97(243) 13–255	WP_049597737.1
	molecular chaperone [*Yersinia frederikseniir*]<br>Length: 255<br>81/90(242) 14–255	WP_032911350.1
PL78_09560 (255)	nicotinamidase [*Salmonella enterica*]<br>Length: 225<br>92/95(225) 1–225	WP_050178696.1
PL78_09565 (303)<br>LysR	hypothetical protein [*Massilia niastensis*]<br>Length: 302<br>74/85(301) 1–301	WP_020656025.1
	LysR family transcriptional regulator [*Aeromonas aquatica*]<br>Length: 306<br>70/82(301) 1–301	WP_033130787.1
PL78_09570 (619)<br>Transporters	potassium transporter KefC [*Yersinia nurmii*]<br>Length: 619<br>98/98(619) 1–619	WP_049597738.1
	potassium transporter KefC [*Citrobacter amalonaticus*]<br>Length: 620<br>86/91(619) 1–619	WP_046475364.1
PL78_09575 (176)<br>Transporters	potassium transporter KefF [*Yersinia nurmii*]<br>Length: 176<br>96/97(176) 1–176	WP_049597739.1
	MULTISPECIES: potassium transporter [*Enterobacteriaceae*]<br>Length: 176<br>88/94(176) 1–176	WP_003018878.1

PL78_09575 (127)	hypothetical protein [*Yersinia nurmii*]<br>Length: 129<br>96/99(129) 1–129	WP_049597740.1
	hypothetical protein [*Yersinia aldovae*]<br>Length: 129<br>73/89(129) 1–129	WP_042546198.1
PL78_09580 (127)	hypothetical protein [*Yersinia nurmii*]<br>Length: 129<br>96/99(127) 3–129	WP_049597740.1
	hypothetical protein [*Yersinia aldovae*]<br>Length: 129<br>72/89(127) 2–129	WP_042546198.1
	hypothetical protein [*Photorhabdus luminescens*]<br>Length: 123<br>50/64(119) 1–119	WP_011148526.1
PL78_09585 (96)	hypothetical protein [*Yersinia nurmii*]<br>Length: 96<br>78/85(91) 1–91	WP_049597741.1
	hypothetical protein [*Yersinia intermedia*]<br>Length: 112<br>52/67(87) 1–87	WP_050074814.1
PL78_09590 (137)<br>PirA	hypothetical protein [*Yersinia intermedia*]<br>Length: 136<br>79/88(137) 1–136	WP_032905692.1
	Uncharacterised protein [*Yersinia nurmii*]<br>Length: 137<br>95/97(137) 1–137	embCNE45801.1

PL78_09595 (416)<br>PirB	hypothetical protein [*Yersinia nurmii*]<br>Length: 416<br>93/96(416) 1–416	WP_049597742.1
	hypothetical protein [*Yersinia intermedia*]<br>Length: 416<br>86/94(416) 1–416	WP_050086266.1
	toxin PirB [*Photorhabdus luminescens*]<br>Length: 419<br>47/66(396) 17–412	WP_046395117.1
PL78_09600 (426)<br>Lipase	thermolabile hemolysin [*Yersinia intermedia*]<br>Length: 426<br>82/91(426) 1–426	WP_050086267.1
	thermolabile hemolysin [*Yersinia nurmii*]<br>Length: 426<br>95/97(426) 1–426<br>pfam00657: Lipase_GDSL	WP_049597743.1
PL78_09605 (255)<br>Iron transporter	iron ABC transporter [*Erwinia toletana*]<br>Length: 255<br>77/86(255) 1–255	WP_026111923.1
PL78_09610 (320)<br>Iron transporter	iron-dicitrate transporter subunit FecD [*Rahnella aquatilis*]<br>Length: 320<br>82/90(320) 1–320	WP_047607211.1
	Fe3+ dicitrate ABC transporter permease [*Rahnella aquatilis*]<br>Length: 320<br>82/90(320) 1–320	WP_014341819.1
PL78_09615 (333)<br>Iron transporter	iron ABC transporter [*Rahnella aquatilis*]<br>Length: 334<br>85/91(333) 1–334	WP_014341818.1
PL78_09620 (302)	iron siderophore-binding protein [*Rahnella aquatilis*]<br>Length: 301<br>80/88(289) 13–301	WP_014341817.1
PL78_09625 (785)<br>Iron transporter	transporter [*Erwinia toletana*]<br>Length: 786<br>83/91(784) 3–786	WP_017802701.1

PL78_09630 (321)<br>Iron transporter	iron dicitrate transport regulator FecR [*Erwinia toletana*]<br>Length: 309<br>69/82(309) 1–309	WP_051050855.1
	iron dicitrate transport regulator FecR [*Rahnella aquatilis*]<br>Length: 323<br>68/81(323) 1–323	WP_047610250.1
PL78_09635 (175)	RNA polymerase sigma factor [*Rahnella aquatilis*]<br>Length: 172<br>81/88(171) 1–171	WP_014341814.1
PL78_09645 (193)	MULTISPECIES: protein RclC [*Escherichia*]<br>Length: 197<br>84/90(186) 1–186	WP_001355150.1
PL78_09650 (441)	pyridine nucleotide-disulfide oxidoreductase [*Escherichia coli*]<br>Length: 441<br>73/85(441) 1–441	WP_038340583.1
PL78_09655 (288)	AraC family transcriptional regulator [*Ewingella americana*]<br>Length: 284<br>61/74(282) 1–282	WP_034790889.1
PL78_09660(500)	glycine/betaine ABC transporter [*Yersinia nurmii*]<br>Length: 500<br>100/100(500) 1–500	WP_049597746.1
	glycine/betaine ABC transporter [*Yersinia frederikseniir*]<br>Length: 500<br>94/98(500) 1–500	WP_050108470.1
PL78_09665 (350)	6-O-methylguanine DNA methyltransferase [*Yersinia nurmii*]<br>Length: 350<br>93/95(349) 1–349	WP_049597747.1
	hypothetical protein [*Serratia marcescens*]<br>Length: 351<br>67/79(343) 11–351	WP_021504722.1

PL78_09670 (200)	DNA-3-methyladenine glycosidase [*Yersinia nurmii*]<br>Length: 200<br>95/97(200) 1–200	WP_049597748.1
	DNA-3-methyladenine glycosidase [*Yersinia rohdei*]<br>Length: 202<br>71/82(200) 1–200	WP_032817263.1
PL78_09675 (501)	peptide synthetase [*Yersinia nurmii*]<br>Length: 501<br>96/98(500) 1–500	WP_049597749.1
	peptide synthetase [*Yersinia ruckeri*]<br>Length: 501<br>76/83(500) 1–500	WP_042525915.1
PL78_09680 (248)	2,3-dihydro-2,3-dihydroxybenzoate dehydrogenase [*Yersinia nurmii*]<br>Length: 248<br>96/98(248) 1–248	WP_049598095.1
	2,3-dihydro-2,3-dihydroxybenzoate dehydrogenase [*Yersinia ruckeri*]<br>Length: 251<br>86/90(251) 1–251	gbKFE37680.1
PL78_09685 (294)	isochorismatase [*Yersinia nurmii*]<br>Length: 294<br>97/97(294) 1–294	WP_049597750.1
	isochorismatase [*Yersinia ruckeri*]<br>Length: 294<br>85/91(294) 1–294	WP_042525917.1
PL78_09690 (543)	enterobactin synthase subunit E [*Yersinia nurmii*]<br>Length: 543<br>97/98(543) 1–543	WP_049597751.1
	enterobactin synthase subunit E [*Yersinia ruckeri*]<br>Length: 544<br>86/91(540) 1–540	WP_038244834.1

PL78_09695 (389)	menaquinone-specific isochorismate synthase [*Yersinia nurmii*]<br>Length: 415<br>96/97(415) 1–415	embCNE46363.1
	Enterobactin synthetase component C (Isochorismate synthase) [*Yersinia ruckeri* ATCC 29473]<br>Length: 402<br>80/87(402) 1–402	gbEEP98231.1
PL78_09700 (346)	iron ABC transporter substrate-binding protein [*Yersinia nurmii*]<br>Length: 349<br>95/96(346) 1–346	WP_049597752.1
	iron ABC transporter substrate-binding protein [*Yersinia ruckeri*]<br>Length: 346<br>77/86(346) 1–346	WP_038244836.1
PL78_09705 (425)	POT family transporter [*Yersinia nurmii*]<br>Length: 425<br>97/98(422) 1–425	WP_049597753.1
	POT family transporter [*Yersinia ruckeri*]<br>Length: 422<br>91/96(422) 1–422	WP_038244838.1
PL78_09710 (312)<br>Iron transporter	iron-enterobactin transporter membrane protein [*Yersinia nurmii*]<br>Length: 351<br>97/98(351) 1–351	embCNE46474.1
	Ferric enterobactin transport protein [*Yersinia ruckeri* ATCC 29473]<br>Length: 351<br>87/94(351) 1–351	gbEEP98234.1
PL78_09715 (347)<br>Iron transporter	iron ABC transporter permease [*Yersinia nurmii*]<br>Length: 347<br>97/97(347) 1–347	WP_049597754.1
	iron-enterobactin transporter permease [*Yersinia enterocolitica*]<br>Length: 347<br>81/89(347) 1–347	WP_019079081.1

PL78_09720 (276)<br>Iron transporter	iron-dicitrate transporter ATP-binding subunit [*Yersinia nurmii*]<br>Length: 276<br>97/98(276) 1–276	WP_049597755.1
	iron-dicitrate transporter ATP-binding subunit [*Yersinia ruckeri*]<br>Length: 275<br>86/94(271) 1–271	WP_045844292.1
PL78_09725 (219)	hypothetical protein [*Yersinia nurmii*]<br>Length: 219<br>85/90(219) 1–219	WP_049597756.1
PL78_09730 (2399)<br>chromophore lyase	chromophore lyase [*Yersinia nurmii*]<br>Length: 2399<br>high molecular weight siderophore biosynthesis protein [*Yersinia nurmii*]<br>Length: 2399<br>96/97(2399) 1–2399	WP_049597757.1<br>emb|CNE46596.1|
	chromophore lyase [*Yersinia ruckeri*]<br>Length: 2386<br>72/83(2400) 1–2384	WP_038244849.1
PL78_09735 (73)	Uncharacterized protein conserved in bacteria [*Yersinia nurmii*]<br>97/98(74) 16–89	embCNE46635.1
	antibiotic synthesis protein MbtH [*Yersinia ruckeri*]<br>Length: 74<br>81/89(74) 1–74	WP_038244851.1
	antibiotic synthesis protein MbtH [*Xenorhabdus cabanillasii*]<br>Length: 84<br>56/67(73) 1–69	WP_051502373.1
PL78_09740 (435)<br>Enterochelin esterase	enterochelin esterase [*Yersinia nurmii*]<br>Length: 435<br>95/97(435) 1–435	WP_049597758.1
	enterochelin esterase [*Yersinia ruckeri*]<br>Length: 439<br>80/87(435) 1–435	WP_042525934.1

PL78_09745 (736)<br>Ferrichrysobactin receptor	ferrichrysobactin receptor [*Yersinia nurmii*]<br>Length: 736<br>98/99(736) 1–736	WP_049597759.1
	ferrichrysobactin receptor [*Yersinia ruckeri*]<br>Length: 735<br>88/94(736) 1–735	WP_004722010.1
PL78_09750 (139)	Uncharacterised protein [*Yersinia nurmii*]<br>Length: 139<br>95/97(119) 21–139	embCNE46726.1
	hypothetical protein [*Yersinia mollaretii*]<br>Length: 136<br>59/69(126)11–136	WP_049646990.1
PL78_09755 (112)	hypothetical protein [*Yersinia nurmii*]<br>Length: 112<br>88/91(112) 1–112	WP_049597761.1
	putative efflux system protein [*Yersinia enterocolitica*]<br>Length: 129<br>55/69(92) 38–128	embCFQ35865.1
PL78_09760 (426)<br>Transporter	heavy metal RND transporter [*Yersinia nurmii*]<br>Length: 426<br>93/95(424) 1–424	WP_049597762.1
	heavy metal RND transporter [*Yersinia intermedia*]<br>Length: 432<br>75/84(432) 1–432	WP_050077138.1
PL78_09765 (503)<br>Transporter	cobalt transporter [*Yersinia nurmii*]<br>Length: 503<br>92/94(503) 1–503	WP_049597763.1
	cobalt transporter [*Yersinia frederikseniir*]<br>Length: 509<br>71/83(509) 1–509	WP_050300177.1

PL78_09770 (1040)<br>Transporter	cation transporter [*Yersinia nurmii*]<br>Length: 1040<br>97/98(1040) 1–1040	WP_049597764.1
	putative cation efflux system protein [Yersinia kristensenii]<br>Length: 1043<br>92/96(1039) 1–1039	embCFR25599.1
PL78_09775 (272)	shikimate dehydrogenase [*Yersinia nurmii*]<br>Length: 272<br>97/98(272)1–272	WP_049597765.1
	shikimate dehydrogenase [*Yersinia ruckeri*]<br>Length: 272<br>88/94(271) 1–271	WP_038244855.1
PL78_09780 (141)<br>Transporter	biopolymer transporter ExbD [*Yersinia nurmii*]<br>Length: 141<br>99/100(141) 1–141	WP_049597766.1
	biopolymer transporter ExbD [*Yersinia ruckeri*]<br>Length: 141<br>91/96(141) 1–141	WP_004720996.1
PL78_09785 (238)	MotA/TolQ/ExbB proton channel family protein [*Yersinia nurmii*]<br>Length: 339<br>97/97(341) 1–339	embCNE47005.1
	tonB-system energizer ExbB [*Yersinia ruckeri*]<br>Length: 339<br>85/90(342) 1–339	gbAJI95450.1
PL78_09790 (402)	cystathionine beta-lyase [*Yersinia nurmii*]<br>Length: 398<br>97/98(398) 1–398	WP_049597767.1
	cystathionine beta-lyase [*Yersinia ruckeri*]<br>Length: 398<br>Rang88/95(394) 1–394	gbAJI94061.1

PL78_09795 (220)	membrane protein [*Yersinia nurmii*]<br>Length: 220<br>99/100(220) 1–220	WP_049597768.1
	membrane protein [*Yersinia ruckeri*]<br>Length: 220<br>92/95(220) 1–220	WP_004721003.1
PL78_09800 (297)	AraC family transcriptional regulator [*Yersinia nurmii*]<br>Length: 297<br>96/96(297) 1–297	WP_049598101.1
	AraC family transcriptional regulator [*Yersinia ruckeri*]<br>Length: 299<br>89/94(297) 1–297	WP_038244888.1

Table S2. *Yersinia entomophaga* Rhs associated regions 2–5. For graphic depiction refer to Figure 3 of the associated manuscript.

Yersinia entomophaga Rhs associated region 2.

Locus (Amino Acid Resides)	Closest BlastP Orthologue [Species] Length: Amino Acid Length of Orthologue % Identity/% Similarity (over the Indicated Range of Amino acid Residues) in Relation to Target Sequence. Protein Domain	Accession Number
PL78_18705(171) Ferritin	ferritin [*Yersinia nurmii*] Length: 171 99/99(171) 1–171	WP_049600522.1
	ferritin [*Yersinia ruckeri*] Length: 171 97/97(171) 1–171	WP_004721727.1
PL78_18710(128)	hypothetical protein [*Yersinia nurmii*] Length: 128 98/100(128) 1–128	WP_049600525.1
	hypothetical protein [*Yersinia ruckeri*] Length: 128 94/99(128) 1–128	WP_038243346.1
PL78_18715 (294) CopD	copper resistance protein CopD [*Yersinia nurmii*] Length: 294 96/97(294) 1–294	WP_049600528.1
	copper resistance protein CopD [*Yersinia ruckeri*] Length: 294 80/87(294) 1–94	WP_045844133.1
PL78_18720 (113)	hypothetical protein [*Yersinia nurmii*] Length: 113 97/98(113) 1–113	WP_049600531.1
	hypothetical protein [*Yersinia mollaretii*] Length: 113 81/90(113) 1–113	WP_004875990.1

PL78_18725 (263)	integrase [*Yersinia enterocolitica*]<br>Length: 369<br>97/98(263) 107–369	WP_050876385.1
PL78_18730 (100)	MULTISPECIES: transposase [*Yersinia*]<br>Length: 100<br>100/100(100) 1–100	WP_042526049.1
PL78_18735 (56)	IS911 orfB [*Shigella flexneri* 2a str. 2457T]<br>Length: 52<br>82/90(51) 1–51	gbAAP16190.1
PL78_18740 (68)	Putative transposase [*Yersinia enterocolitica*]<br>Length: 138<br>93/95(68) 71–138	embCFB70873.1
PL78_18745(155)	hypothetical protein [*Yersinia ruckeri*]<br>Length: 542<br>99/98(253) 1–153	WP_038246006.1
PL78_18750 (220)	MULTISPECIES: hypothetical protein [*Serratia*]<br>Length: 261<br>60/78(218) 44–260	WP_025122879.1
PL78_18755(91)	hypothetical protein [*Yersinia aldovae*]<br>Length: 105<br>49/69(89) 17–105	WP_042546108.1
PL78_18760 (207)<br>LopT	hypothetical protein [*Yersinia pekkanenii*]<br>Length: 221<br>77/86(195) 12–206	WP_049615575.1
	cysteine protease [*Photorhabdus luminescens*]<br>Length: 323<br>24/40(181) 131–297	WP_058589369.1
PL78_18765 (105)	hypothetical protein [*Yersinia nurmii*]<br>Length: 105<br>92/96(104) 1–105	WP_049600537.1
	hypothetical protein [*Yersinia aldovae*]<br>Length: 105<br>59/71(104) 1–105	WP_042546108.1

PL78_18770 (95)	hypothetical protein [*Yersinia aldovae*]<br>Length: 268<br>61/74(94) 44–136	WP_042546107.1
PL78_18775 (120)	transposase [*Yersinia rohdei*]<br>Length: 197<br>46/53(167) 1–167	embCNF45791.1
PL78_18780 (965)<br>RhsA	rhsa toxin protein [*Yersinia nurmii*]<br>Length: 963<br>87/90(965) 1–963	embCNE97220.1
	insecticidal toxin complex protein TccC3 [*Vibrio parahaemolyticus*] (tc_PAI_042)<br>Length: 971<br>76/83(971) 1–971	gbAHZ10944.1
PL78_18785 (458)	hypothetical protein [*Photorhabdus luminescens*]<br>Length: 462<br>45/64(457) 6–460	WP_049582883.1
	hypothetical protein [*Yersinia nurmii*]<br>Length: 458<br>94/96(458) 1–458	WP_049600554.1
PL78_18790 (150)<br>Ivy	inhibitor of vertebrate lysozyme precursor [*Salmonella enterica* subsp. VII]<br>Length: 155<br>69/84(150) 6–155	embCAX67957.1
	Probable inhibitor of vertebrate lysozyme [*Erwinia tasmaniensis* Et1/99]<br>Length: 154<br>62/76(150) 7–154	embCAO95555.1
PL78_18795(205)<br>LyseE	lyseE threonine transporter [*Raoultella terrigena*]<br>Length: 205<br>84/91(205) 1–205	WP_045857255.1
PL78_18800(182)	acetyltransferase [*Yersinia ruckeri*]<br>Length: 182<br>92/98(182) 1–182	WP_038244025.1

Yersinia entomophaga Rhs associated region 3.

**Locus (Amino Acid Residues)**	**Closest BlastP Orthologue [Species]**<br>**Length: Amino Acid Length of Orthologue**<br>**% Identity/% Similarity (over the Indicated Range of Amino Acid Residues) in Relation to Target Sequence.**<br>**Protein Domain**	**Accession Number**
PL78_00880 (249)	transposase [*Yersinia ruckeri*]<br>Length: 249<br>99/100 (249) 1–249	WP_042527218.1
PL78_00885 (88)	transposase [*Yersinia enterocolitica*]<br>Length: 96<br>100/100(88) 9–96	WP_011815814.1
PL78_00890 (252)	IS1329 transposase B [*Yersinia enterocolitica*]<br>Length: 280<br>89/93(207) 1–207	embCRX47735.1
PL78_00895 (187)	hypothetical protein KOXM_28035 [*Klebsiella oxytoca* M5al]<br>Length 116<br>87/93(187) 1–187	gbEKP24881.1
	MULTISPECIES: membrane protein [*Enterobacteriaceae*]<br>Length: 187<br>87/93(87) 1–187	WP_024358370.1
	hypothetical protein [Serratia sp. Leaf51]<br>Length: 187<br>94/97(187) 1–187	WP_056771403.1
PL78_00900 (172)<br>Hcp	major exported protein [*Yersinia nurmii*]<br>Length: 172<br>100/100(172) 1–172	WP_049602039.1
	hypothetical protein [*Photorhabdus temperata*]<br>Length: 172<br>95/97(172) 1–172	WP_021323277.1

PL78_00905 (165)	type VI secretion protein [*Yersinia nurmii*]<br>Length: 165<br>99/99(165) 1–165<br>"type VI secretion protein"	WP_049602042.1
PL78_00910 (493)<br>ImpC	type VI secretion protein [*Yersinia nurmii*]<br>Length: 493<br>100/100(493) 1–493	WP_049602045.1
	MULTISPECIES: type VI secretion protein [*Yersinia pseudotuberculosis* complex]<br>Length: 493<br>93/96(493) 1–493	WP_025381701.1
PL78_00915 (146)<br>Lys	lysozyme [*Yersinia nurmii*]<br>Length: 146<br>98/98(146) 1–146	WP_049602048.1
	MULTISPECIES: lysozyme [Serratia]<br>Length: 146<br>70/84(145) 1–146	WP_025123010.1
PL78_00920 (602)<br>ImpG	type VI secretion protein [*Yersinia nurmii*]<br>Length: 602<br>99/99(602) 1–602	WP_049602051.1
	type VI secretion protein [*Yersinia pekkanenii*]<br>Length: 606<br>80/89(606) 1–602	WP_049612770.1
PL78_00925(347)	type VI secretion protein [*Yersinia nurmii*]<br>Length: 347<br>100/100(335) 1–347	WP_049602054.1
	type VI secretion protein [*Yersinia pseudotuberculosis*]<br>Length: 362<br>80/90(335) 1–347	WP_050136839.1
PL78_00930 (436)	phosphopeptide-binding protein [*Yersinia nurmii*]<br>Length: 436<br>99/99(436) 1–436	WP_049602058.1
	MULTISPECIES: phosphopeptide-binding protein [*Yersinia pseudotuberculosis* complex]<br>Length: 438<br>67/79(439) 1–436	WP_033852633.1

PL78_00935 (180)	type VI secretion protein [*Yersinia nurmii*]<br>Length: 180<br>98/99(180) 1–180	WP_049602061.1
	type VI secretion protein [*Xenorhabdus khoisanae*]<br>Length: 184<br>66/80(184) 1–180	WP_047964021.1
PL78_00940 (448)<br>ImpJ	type VI secretion protein [*Yersinia nurmii*]<br>Length: 448<br>99/99(448) 1–444	WP_049602062.1
	type VI secretion protein [*Yersinia aldovae*]<br>Length: 448<br>83/91(448) 1–444	WP_049689231.1
PL78_00945 (255)<br>MotB	membrane protein [*Yersinia nurmii*]<br>Length: 255<br>97/98(255) 1–255	WP_049602063.1
	chemotaxis protein MotB [*Photorhabdus asymbiotica*]<br>Length: 256<br>79/88(256) 1–256	WP_012776473.1
PL78_00950 (866)<br>ClpV	Clp protease ClpV [*Yersinia nurmii*]<br>Length: 866<br>98/99(866) 1–866	WP_049602066.1
	MULTISPECIES: Clp protease ClpV [*Yersinia pseudotuberculosis* complex]<br>Length: 867<br>78/88(867) 1–866	WP_025381694.1
PL78_00955 (266)<br>Fis	Fis family transcriptional regulator [*Yersinia nurmii*]<br>Length: 266<br>96/98(266) 1–266	WP_049602068.1
	Fis family transcriptional regulator [*Yersinia aldovae*]<br>Length: 265<br>71/83(266) 1–265	WP_042546926.1

PL78_00960 (218)	type VI secretion protein [*Yersinia nurmii*]<br>Length: 218<br>96/96(218) 1–218	WP_049602071.1
	type VI secretion protein [*Yersinia aldovae*]<br>Length: 219<br>63/73(192) 30–219	WP_042546925.1
PL78_00965 (470)<br>ImpA	membrane protein [*Yersinia nurmii*]<br>Length: 467<br>96/96(467) 1–467	WP_049602074.1
	MULTISPECIES: membrane protein [Serratia]<br>Length: 462<br>60/73(471)1–467	WP_025123019.1
PL78_00970 (1183)<br>IcmF<br>Orf3	type VI secretion protein IcmF [*Yersinia nurmii*]<br>Length: 1183<br>98/98(1183) 1–1183	WP_049602076.1
	lipoprotein [*Yersinia pseudotuberculosis*]<br>Length: 1177<br>73/85(1176) 2–177	embCFV32787.1
	type VI secretion protein IcmF [*Photorhabdus luminescens*]<br>Length: 1181<br>66/80(1168) 16–1181	WP_011144748.1
PL78_00975 (441)<br>ImpA	membrane protein [*Yersinia nurmii*]<br>Length: 441<br>97/98(441) 1–441	WP_049602079.1
	membrane protein [*Yersinia aldovae*]<br>Length: 442<br>58/73(443) 1–441	WP_049634662.1
	hypothetical protein [*Photorhabdus luminescens*]<br>Length: 459<br>34/51(466) 1–454	WP_011148302.1

PL78_00980 (770) VgR	type VI secretion protein Vgr [*Yersinia nurmii*]<br>Length: 756<br>96/96(770) 1 to 756	WP_049602081.1
	type VI secretion protein Vgr [*Serratia fonticola*]<br>Length: 743<br>72/80(705) 52–743	WP_037413087.1
	unnamed protein product [*Photorhabdus luminescens* subsp. laumondii TTO1]<br>Length: 704<br>39/57(612) 3–604	embCAE12757.1
PL78_00985 (139) Orf5	hypothetical protein [*Yersinia nurmii*]<br>Length: 139<br>98/98(138) 1–138	WP_049602084.1
	hypothetical protein [*Photorhabdus luminescens*]<br>Length: 139<br>64/79(139) 1–139	WP_049584104.1
PL78_00990 (1444) Rhs	YD repeat-/RHS repeat-containing protein [*Yersinia nurmii*]<br>Length: 1451<br>99/99(1320) 1–1320	embCNF22828.1
	type IV secretion protein Rhs [*Photorhabdus asymbiotica*]<br>Length: 1451<br>59/72(1345) 1–1320	WP_012777142.1
PL78_00995 (123) Spt4	hypothetical protein [*Yersinia nurmii*]<br>Length: 123<br>98/99(123) 1–123	WP_049602103.1
	hypothetical protein [*Leminorella grimontii*]<br>Length: 123<br>100/100(13) 1–123<br>COG2093: Spt4	WP_027272928.1

PL78_01000 (117)	MULTISPECIES: hypothetical protein [*Yersinia pseudotuberculosis* complex]<br>Length: 117<br>85/93(117) 1–117	WP_025381673.1
	Uncharacterised protein [*Yersinia similis*]<br>Length: 117<br>85/92(117) 1–117	WP_054878057.1
	hypothetical protein [*Photorhabdus luminescens*]<br>Length: 115<br>66/84(117) 1–115	WP_011147332.1
PL78_01005 (145)	type IV secretion protein Rhs [*Pectobacterium carotovorum*]<br>Length: 1424<br>83/89(144) 1280–1423	WP_039503594.1
	hypothetical protein SK41_01880 [*Enterobacter aerogenes*]<br>Length: 255<br>79/86(145) 111–254	gbKLV93059.1
PL78_01010 (147)	hypothetical protein [*Pectobacterium wasabiae*]<br>Length: 145<br>61/64(142) 1–141	WP_025919449.1
	hypothetical protein [*Enterobacter cloacae*]<br>Length: 141<br>60/78(132) 10–140	WP_047174021.1
PL78_01015 (67)	hypothetical protein [*Yersinia nurmii*]<br>Length: 67<br>100/100(67) 1–67	WP_049602119.1
	hypothetical protein [Morganella morganii]<br>Length: 284<br>75/95(67) 218–284	WP_052927290.1
PL78_01025 (159)	hypothetical protein [Lonsdalea quercina]<br>Length: 159<br>94/96(159) 1–149	WP_036150158.1
PL78_01030 (97)<br>TIGR03696: Rhs_assc_core	type IV secretion protein Rhs [Pantoea ananatis]<br>Length: 1395<br>72/83(99) 1260–1358	WP_028723925.1

PL78_01035 (82)	hypothetical protein [*Yersinia nurmii*]<br>Length: 82<br>98/100(82) 1–82	WP_049602116.1
	hypothetical protein [*Pantoea* sp. BL1]<br>Length: 82<br>59/81(82) 1–82	WP_045834389.1
	hypothetical protein [Type-F symbiont of *Plautia stali*]<br>Length: 82<br>59/82(82) 1–82	WP_058958422.1
PL78_01040 (139)<br>AHH<br>pfam14412	hypothetical protein [*Yersinia nurmii*]<br>Length: 127<br>97/98(122) 6–127	WP_053215256.1
	hypothetical protein [*Serratia fonticola*]<br>Length: 138<br>98/99(138) 1–138	WP_051363166.1
	hypothetical protein ECA4293 [*Pectobacterium atrosepticum* SCRI1043]<br>Length: 150<br>74/83(139) 12–150	embCAG77190.1
	type IV secretion protein Rhs [*Serratia fonticola*]<br>Length: 1487<br>99/99(139) 1349–1487	WP_046808161.1
PL78_01045 (173)	hypothetical protein [*Serratia fonticola*]<br>Length: 173<br>99/99(173) 1–173	P_046808162.1
	hypothetical protein [*Yersinia nurmii*]<br>Length: 173<br>95/97(173) 1–173	WP_049596802.1
	hypothetical protein [*Photorhabdus luminescens*]<br>Length: 177<br>34/57(173) 5–174	WP_036812017.1
PL78_01050 (321)	membrane protein [*Yersinia nurmii*]<br>Length: 321<br>98/99(284) 38–321	WP_049596801.1
	putative membrane protein [*Yersinia intermedia*]<br>Length: 284<br>83/93(284) 1–284	gbAJJ19515.1

Yersinia entomophaga Rhs associated region 4.

**Locus (Amino Acid Residues)**	**Closest BlastP Orthologue [Species]**<br>**Length: Amino Acid Length of Orthologue**<br>**% Identity/% Similarity (over the Indicated Range of Amino Acid Residues) in Relation to Target Sequence.**<br>**Protein Domain**	**Accession Number**
PL78_11955 (317)	ketodeoxygluconokinase [*Yersinia nurmii*]<br>Length: 317<br>95/96(317) 1–317	WP_049597328.1
	ketodeoxygluconokinase [*Yersinia ruckeri*]<br>Length: 317<br>86/92(315) 1–315	WP_049689687.1
PL78_11960 (515)	peptidase [*Yersinia nurmii*]<br>Length: 515<br>96/97(515) 1–515	WP_049597329.1
	peptidase [*Yersinia ruckeri*]<br>Length: 496<br>90/94(490) 1–490	WP_042526849.1
PL78_11965 (430)	C4-dicarboxylate transporter [*Yersinia nurmii*]<br>Length: 430<br>99/99(430) 1–430	WP_049597330.1
	C4-dicarboxylate ABC transporter [*Yersinia ruckeri*]<br>Length: 430<br>97/98(430) 1–430	WP_004723244.1
PL78_11970 (655)	biofilm formation regulator HmsP [*Yersinia nurmii*]<br>Length: 666<br>98/99(655) 12–666	embCNE26472.1
	biofilm formation regulator HmsP [*Yersinia ruckeri*]<br>Length: 655<br>81/90(655) 1–655	WP_038242966.1

PL78_11975 (1159)<br>CelC	cellulose synthase [*Yersinia ruckeri*]<br>Length: 1160<br>86/92(1160) 1–1160	WP_042526844.1
	cellulose synthase [*Yersinia ruckeri*]<br>Length: 1160<br>86/92(1160) 1–1160	WP_038242904.1
PL78_11980 (377)<br>CelD	endoglucanase [*Yersinia nurmii*]<br>Length: 377<br>97/97(377) 1–377	WP_049597332.1
	endoglucanase [*Yersinia ruckeri*]<br>Length: 376<br>86/91(377) 1–376	WP_004723238.1
PL78_11985 (770)<br>CelB	cellulose synthase [*Yersinia nurmii*]<br>Length: 770<br>96/97(770) 1–770	WP_049597464.1
	cellulose synthase [*Yersinia ruckeri*]<br>Length: 770<br>90/94(770) 1–770	WP_042528353.1
PL78_11990 (875)<br>CelA	cellulose synthase [*Yersinia nurmii*]<br>Length: 875<br>98/98(875) 1–875	WP_049597333.1
	cellulose synthase [*Yersinia ruckeri*]<br>Length: 875<br>91/95(875) 1–875	WP_038276495.1
PL78_11995 (243)<br>CelT	cell division protein [*Yersinia nurmii*]<br>Length: 243<br>95/98(243) 1–243	WP_049597334.1
	cell division protein [*Yersinia ruckeri*]<br>Length: 245<br>79/87(245) 1–245	WP_038276497.1

PL78_12000 (63)<br>CelS	hypothetical protein [*Yersinia nurmii*]<br>Length: 63<br>90/92(63) 1–63	WP_049597335.1
	hypothetical protein [*Yersinia ruckeri*]<br>Length: 63<br>90/95(63) 1–63	WP_038242910.1
PL78_12005 (518)<br>CelP	cellulose biosynthesis protein BcsE [*Yersinia ruckeri*]<br>Length: 518<br>84/92(518) 1–518	WP_042526838.1
WP_042526824.1	WP_042526824.1	WP_042526824.1
	membrane protein [*Yersinia ruckeri*]<br>Length: 546<br>84/91(553) 1–546	WP_042526834.1
PL78_12020 (334)	dipeptide transporter ATP-binding subunit [*Yersinia nurmii*]<br>Length: 341<br>98/98(334) 8–334	embCNE26976.1
	dipeptide transporter ATP-binding subunit [*Yersinia enterocolitica*]<br>Length: 341<br>98/98(333) 8–340	embCFQ43661.1
PL78_12025 (326)	peptide ABC transporter ATP-binding protein [*Yersinia nurmii*]<br>Length: 326<br>99/100(1–326) 1–326	WP_049597338.1
	peptide ABC transporter ATP-binding protein [*Yersinia ruckeri*]<br>Length: 326<br>98/98(326) 1–326	WP_042526832.1
PL78_12030 (300)	peptide transporter [*Yersinia nurmii*]<br>Length: 300<br>99/100(300) 1–300	WP_049597339.1
	peptide ABC transporter [*Yersinia ruckeri*]<br>Length: 300<br>98/99(300) 1–300	WP_004718359.1

PL78_12035 (339)	hypothetical protein [*Yersinia nurmii*]<br>Length: 339<br>99/99(339) 1–339	WP_049597340.1
	hypothetical protein [*Yersinia ruckeri*]<br>Length: 339<br>99/99(33) 1–339	WP_038242913.1
PL78_12040 (536)	peptide ABC transporter substrate-binding protein [*Yersinia nurmii*]<br>Length: 536<br>99/99(536) 1–536	WP_049597341.1
	peptide ABC transporter substrate-binding protein [*Yersinia ruckeri*]<br>Length: 536<br>97/98(536) 1–536	WP_004718354.1
PL78_12050 (443)	MFS transporter [*Yersinia nurmii*]<br>Length: 443<br>99/100(443) 1–443	WP_049597342.1
	hypothetical protein [*Yersinia ruckeri*]<br>Length: 445<br>97/98(443) 1–443	WP_004718352.1
PL78_12045 tRNA-Pro		
PL78_12050 (443)	MFS transporter [*Yersinia nurmii*]<br>Length: 443<br>99/100(443) 1–443	WP_049597342.1
	hypothetical protein [*Yersinia ruckeri*]<br>Length: 445<br>97/98(443) 1–443	WP_004718352.1
PL78_12055 (509)	histidine kinase [*Yersinia nurmii*]<br>Length: 509<br>99/99(509) 1–509	WP_049597343.1
	histidine kinase [*Yersinia ruckeri*]<br>Length: 509<br>91/95(509) 1–509	WP_038242914.1

PL78_12060 (196)	DNA-binding response regulator [*Yersinia nurmii*]<br>Length: 196<br>99/99(196) 1–196	WP_049597344.1
	DNA-binding response regulator [*Yersinia ruckeri*]<br>Length: 196<br>95/98(196) 1–196	WP_038242915.1
PL78_12065 (563)	phosphoethanolamine transferase [*Yersinia nurmii*]<br>Length: 563<br>98/99(563) 1–563	WP_049597345.1
	phosphoethanolamine transferase [*Yersinia ruckeri*]<br>Length: 563<br>91/95(561) 1–561	WP_042528351.1
PL78_12070 (141)	organic hydroperoxide resistance protein [*Yersinia nurmii*]<br>Length: 141<br>98/98(141) 1–141	WP_049597346.1
	MULTISPECIES: organic hydroperoxide resistance protein [*Yersinia*]<br>Length: 141<br>95/91(1410) 1–141	WP_038637498.1
PL78_12075 (149)	homoprotocatechuate degradative operon repressor [*Yersinia nurmii*]<br>Length: 144<br>95/97(144) 1–144	embCNE27397.1
	Transcriptional regulator, MarR family [*Yersinia frederikseniir* ATCC 33641]<br>Length: 144<br>85/93(143) 1–143	gbEEQ15729.1
PL78_12080 (487)	membrane protein [*Yersinia nurmii*]<br>Length: 487<br>98/99(487) 1–487	WP_049597348.1
	membrane protein [*Yersinia ruckeri*]<br>Length: 487<br>93/96(487) 1–497	WP_038242916.1

PL78_12085 (175)	membrane protein [*Yersinia nurmii*]<br>Length: 175<br>97/97(175) 1–175	WP_049597349.1
	membrane protein [*Yersinia ruckeri*]<br>Length<br>84/90(172) 1–172	WP_004718344.1
PL78_12090 (464	proline-specific permease [*Yersinia nurmii*]<br>Length: 464<br>99/100 1–464	WP_049597350.1
	proline-specific permease [*Yersinia ruckeri*]<br>Length: 464<br>95/97(463) 1–463	WP_042526824.1
PL78_12095 (511)	histidine ammonia-lyase [*Yersinia nurmii*]<br>Length: 511<br>98/98(511) 1–511	WP_049597351.1
	histidine ammonia-lyase [*Yersinia ruckeri*]<br>Length: 511<br>95/97(510) 1–510	WP_038276502.1
PL78_12100 (563)<br>Uhyd	urocanate hydratase [*Yersinia nurmii*]<br>Length: 563<br>99/99(562) 1–562	WP_049597352.1
	urocanate hydratase [*Yersinia ruckeri*]<br>Length: 563<br>98/99(563) 1–563	WP_038242919.1
PL78_12105 (592)<br>IcmF	hypothetical protein [*Yersinia nurmii*]<br>Length: 592<br>90/94(592) 1–592	WP_049597353.1
	type VI secretion protein IcmF [*Pantoea* sp. MBLJ3]<br>Length: 1145<br>32/50(560) 409–950	WP_039659128.1

PL78_12110 (205)<br>VasF	hypothetical protein [*Yersinia nurmii*]<br>Length: 207<br>90/96(196) 6–201	WP_049597354.1
	hypothetical protein [*Yersinia mollaretii*]<br>Length: 207<br>39/60(207) 1–198	WP_032813416.1
PL78_12115 (176)	hypothetical protein [*Serratia fonticola*]<br>Length: 176<br>86/91(175) 1–175	WP_037411404.1
	hypothetical protein [*Xenorhabdus cabanillasii*]<br>Length: 164<br>40–58(172) 1–162	WP_038263077.1
	hypothetical protein [*Photorhabdus luminescens*]<br>Length: 164<br>38/58(170) 1–160	WP_040154291.1
PL78_12120 (131)	hypothetical protein [*Yersinia pseudotuberculosis*]<br>Length: 131<br>88/94(131) 1–131	WP_0503212031
PL78_12125(138)<br>T4SS	type IV secretion protein Rhs [*Yersinia similis*]<br>Length: 457<br>92/96(132) 326–457	gbAHK18882.1
PL78_12130(204)<br>T3SS	type III secretion system effector protein, partial [*Xanthomonas campestris* pv. musacearum NCPPB 4379]<br>locus, KWO_0111025<br>Length: 82<br>37/46(52) 18–69	gbKFA09649.1
	hypothetical protein [*Photorhabdus luminescens*]<br>Length: 201<br>46/56(203) 4–201	WP_049584090.1

PL78_12135 (1400)<br>Rhs	RHS/YD repeat-containing protein [*Yersinia nurmii*]<br>Length: 1395<br>95/96(1257) 1–1297	embCND85328.1
	RHS/YD repeat-containing protein wapA_1 [*Yersinia pseudotuberculosis*]<br>Length: 1406<br>86/90(1297) 1–1297	embCNB75225.1
PL78_12140 (144)	hypothetical protein [*Yersinia nurmii*]<br>Length: 144<br>97/98(144) 1–144	WP_049596388.1
	hypothetical protein [*Serratia fonticola*]<br>Length: 144<br>84/88(144) 1–144	WP_021181280.1
	hypothetical protein [*Photorhabdus luminescens*]<br>Length: 141<br>53/71(135) 6–140	WP_049583043.1
PL78_12145 (249)	integrase core domain protein [*Yersinia ruckeri*]<br>Length: 264<br>99/99(249) 16–264	gbAJI95622.1
	IS1400 transposase B [*Yersinia enterocolitica* subsp. enterocolitica WA-314]<br>Length: 249<br>97/98(249) 1–249	gbEKA28335.1
PL78_12150 (89)	transposase [*Yersinia enterocolitica*]<br>Length: 96<br>100/100(88) 9–96	WP_011815814.1
	transposase [*Citrobacter youngae* ATCC 29220]<br>Length: 95<br>100/100(88) 8–95	gbEFE06341.1

PL78_12155 (110)	aldehyde dehydrogenase [*Yersinia enterocolitica*]<br>Length: 110<br>91/94(110) 1–110	WP_050130145.1
PL78_12160 (141)	hypothetical protein [*Yersinia massiliensis*]<br>Length: 141<br>99/98(141) 1–141	WP_049607925.1
	hypothetical protein [*Photorhabdus luminescens*]<br>Length: 141<br>48/78(141) 1–141	WP_049583905.1
PL78_12165 (619)	helicase [*Yersinia massiliensis*]<br>Length: 619<br>99/99(619) 1–619	WP_049607923.1

Yersinia entomophaga Rhs associated region 5.

Locus (Amino Acid Residues)	Closest BlastP Orthologue [Species]<br>Length: Amino Acid Length of Orthologue<br>% Identity/% Similarity (over the Indicated Range of Amino Acid Residues) in Relation to Target Sequence.<br>Protein Domain	Accession Number
PL78_15035(306)<br>LysR	LysR family transcriptional regulator [*Yersinia nurmii*]<br>Length: 306<br>97/98(306) 1–306	WP_049602006.1
	LysR family transcriptional regulator [*Yersinia ruckeri*]<br>Length: 306<br>94/96(306) 1–306	WP_004721488.1
PL78_15040 (115)	hypothetical protein [*Yersinia nurmii*]<br>Length: 293<br>57/68(89) 186–274	WP_049602009.1
	hypothetical protein [*Yersinia frederikseniir*]<br>Length: 291<br>37/58(114) 161–274	WP_050083633.1
PL78_15045 (93)	hypothetical protein [*Chromobacterium violaceum*]<br>Length: 89<br>47/67(86) 1–86	WP_011135095.1
	hypothetical protein [*Photorhabdus luminescens*]<br>Length: 81<br>45/64(77) 1–77	WP_036775687.1
PL78_15050 (265)	hypothetical protein [*Chromobacterium violaceum*]<br>Length: 265<br>39/59(268) 1–264	WP_011135094.1
PL78_15055 (416)<br>3-o-ACP	3-oxoacyl-ACP synthase [*Chromobacterium violaceum*]<br>Length: 416<br>76/83(416) 1–416	WP_045050403.1

PL78_15060 (321)	unnamed protein product [*Photorhabdus luminescens* subsp. laumondii TTO1]<br>Length: 340<br>28/21(351) 9–329	embCAE14514.1
PL78_15065 (181)	hypothetical protein [*Photorhabdus luminescens*]<br>Length: 186<br>32/52(170) 14–179	WP_049581660.1
	PREDICTED: xaa-Pro dipeptidase isoform X1 [Xenopus (Silurana) tropicalis]<br>Length: 554<br>25/42(146) 121–265	XP_012816407.1
PL78_15070 (1584)<br>Rhs	type IV secretion protein Rhs [Pseudomonas fluorescens]<br>Length: 1577<br>40/58(1449) 1–1433	WP_034134568.1
PL78_15075 (160)<br>NusG	hypothetical protein [*Yersinia intermedia*]<br>Length: 160<br>58/76(159) 1–159	WP_050074372.1
	NusG-type transcription antiterminator [Serratia entomophila]<br>Length: 160<br>53/73(159) 1–159	WP_010895762.1
PL78_15080 (214)<br>AmpC	CMY/LAT/MOX/ACT/MIR/FOX family class C beta-lactamase [*Yersinia ruckeri*]<br>Length: 381<br>96/97(212) 1–212	WP_004721718.1
PL78_15085 (92)	ISEhe3 transposase A [*Yersinia ruckeri* ATCC 29473]<br>Length: 94<br>95/96(92) 3–94	gbEEP97487.1
PL78_15090 (283)	transposase [*Yersinia pekkanenii*]<br>Length: 283<br>99/99(283) 1–283	WP_049615490.1
PL78_15095 (283)	MULTISPECIES: hypothetical protein [*Enterobacteriaceae*]<br>Length: 283<br>100/100(283) 1–283	WP_048990909.1

PL78_15100 (68)	hypothetical protein [*Escherichia coli*]<br>Length: 68<br>100/100(68)1–68	WP_001071601.1
	DNA binding protein [*Plautia stali symbiont*]<br>Length: 70<br>99/100(68) 3–70	dbjBAN95392.1
PL78_15105 (169)	hypothetical protein [*Escherichia coli*]<br>Length: 169<br>96/97(133) 1–133	WP_001610849.1
PL78_15110 (189)	MULTISPECIES: hypothetical protein [*Enterobacteriaceae*]<br>Length: 189<br>97/98(189) 1–189	WP_001610847.1
PL78_15115 (90)	hypothetical protein E1470_c27270 [*Escherichia coli* ECC-1470]<br>Length: 92<br>100//100(90) 3–92	gbAJG09638.1
	His-Xaa-Ser system protein HxsD [*Photorhabdus luminescens*]<br>Length: 90<br>96/98(90)1–90	WP_058588807.1
PL78_15120 (462)	His-Xaa-Ser system radical SAM maturase HxsB [*Escherichia coli*]<br>Length: 462<br>99/100(462) 1–462	WP_023567064.1

Table S3. *Yersinia entomophaga* Type II and III associated regions. For graphic depiction refer to Figure 4 of the associated manuscript.

Yersinia entomophaga T3SSYE1.

Locus (Amino Acid Resides)	Closest BlastP Orthologue [Species] Length: Amino Acid Length of Orthologue % Identity/% Similarity (over the Indicated Range of Amino Acid Residues) in Relation to Target Sequence. Protein Domain	Accession Number
PL78_18070 (296) LuxR	hypothetical protein SLIQ_15070 [Serratia liquefaciens FK01]<br>Length: 209<br>33/55(202) 5–206	dbjGAK27997.1
	hypothetical protein [*Yersinia nurmii*]<br>Length: 296<br>85/91(296) 1–296	WP_049596354.1
PL78_18075 (2347) Intimim	hypothetical protein [*Yersinia nurmii*]<br>Length: 2359<br>92/94(2337) 1–2336	WP_049596353.1
	hypothetical protein [*Hafnia alvei*]<br>Length: 2332<br>44/61(2355) 1–2319	WP_051874183.1
	invasin [*Xenorhabdus bovienii*]<br>Length: 2217<br>43/60(1607) 16–1595	WP_012988596.1
	invasin [*Sodalis praecaptivus*]<br>Length: 934<br>46/53(900) 17–905	WP_025423285.1
PL78_18080 (151)	hypothetical protein [*Yersinia nurmii*]<br>Length: 151<br>79/86(151) 1–151	WP_049596352.1
	alpha-N-arabinofuranosidase [*Cellvibrio japonicus*]<br>Length: 375<br>27/47(100) 93–176	WP_012488648.1

PL78_18085 (241)	hypothetical protein [*Yersinia frederikseniir*]<br>Length: 345<br>33/51(129) 210–316	WP_050318901.1
	hypothetical protein [*Yersinia nurmii*]<br>Length: 241<br>75/87(160) 82–241	WP_049596351.1
PL78_18090 (371)	DUF218 domain [*Yersinia nurmii*]<br>Length: 455<br>93/95(455) 1–455	embCND83898.1
	hypothetical protein [Pseudomonas sp. UW4]<br>Length: 435<br>54/70(391) 44–433	WP_015095362.1
PL78_18095 (139)<br>IaGB	invasion protein IagB [*Yersinia nurmii*]<br>Length: 143<br>97/98(143) 1–143	WP_049596806.1
	invasion protein IagB [*Serratia fonticola*]<br>Length: 146<br>64/80(136) 4–139	WP_046808476.1
PL78_18100 (69)	NA	
PL78_18105 (193)<br>InvF	hypothetical protein [*Serratia fonticola*]<br>Length: 263<br>45/62(264) 1–263)	WP_024486144.1
	hypothetical protein [*Yersinia nurmii*]<br>Length: 255<br>96/98(255) 1–255	WP_049596350.1
PL78_18110 (549)<br>InvG	type III secretion system outer membrane pore InvG [*Serratia fonticola*]<br>Length: 564<br>72/84(564) 1–563	WP_024486143.1
	type III secretion system protein [*Yersinia nurmii*]<br>Length: 562<br>99/99(562) 1–562 1–562	embCND83797.1

PL78_18115 (367)<br>InvE	hypothetical protein [*Yersinia nurmii*]<br>Length: 381<br>96/97(380) 1–380	WP_049596349.1
	hypothetical protein [*Serratia fonticola*]<br>Length: 381<br>64/80(332) 35–366	WP_024486142.1
PL78_18120 (681)<br>InvA	type III secretion system protein InvA [*Yersinia nurmii*]<br>Length: 682<br>98/98(682) 1–682	WP_049596348.1
	type III secretion system protein InvA [*Serratia fonticola*]<br>Length: 685<br>76/88(685) 1–685	WP_024486141.1
PL78_18125 (133)<br>SpaK	hypothetical protein [*Serratia fonticola*]<br>Length: 134<br>59/81(133) 2–134	WP_024486140.1
	hypothetical protein [*Yersinia nurmii*]<br>Length: 133<br>97/100(133) 1–133	WP_049596347.1
PL78_18130 (430)<br>InvC<br>ATP synthase	ATP synthase [*Serratia fonticola*]<br>Length: 430<br>81/88(430) 1–430	WP_024486139.1
	ATP synthase [*Yersinia nurmii*]<br>Length: 430<br>97/98(430) 1–430	WP_049596346.1
PL78_18135 (151)<br>SpaM	hypothetical protein [*Serratia fonticola*]<br>Length: 151<br>44/66(151) 1–151	WP_037413054.1
	hypothetical protein [*Yersinia nurmii*]<br>Length: 151<br>92/94(151) 1–151	WP_049596345.1

PL78_18140 (358)<br>SpaN	hypothetical protein [*Serratia fonticola*]<br>Length: 328<br>39/55(84) 254–328	WP_024486137.1
	hypothetical protein [*Yersinia nurmii*]<br>Length: 358<br>87/91(358) 1–358	WP_049596344.1
PL78_18145 (317)<br>SpaO	hypothetical protein [*Serratia fonticola*]<br>Length: 286<br>51/68(289) 1–286	WP_037413052.1
	hypothetical protein [*Yersinia nurmii*]<br>Length: 317<br>91/96(317) 1–317	WP_049596343.1
PL78_18150(219)<br>FilP	type III secretion system protein [*Yersinia nurmii*]<br>Length: 219<br>99/100(219)1–219	WP_049596342.1
PL78_18155(84)<br>SpaQ	type III secretion system protein SpaQ [*Yersinia nurmii*]<br>Length: 84<br>100/100(84) 1–84	WP_049596341.1
	surface presentation of antigens protein SpaQ [*Cedecea davisae*]<br>Length: 85<br>83/93(83) 1–83	WP_016537922.1
PL78_18160 (254)<br>SpaR	hypothetical protein [*Serratia fonticola*]<br>Length: 254<br>76/85(253) 1–253	WP_024486133.1
	type III secretion apparatus SpaR [*Yersinia nurmii*]<br>Length: 254<br>97/99(254) 1–254	WP_049596340.1
PL78_18165 (350)<br>SpaS	type III secretion system protein SpaS [*Serratia fonticola*]<br>Length: 355<br>72/88(346) 1–346	WP_046808151.1
	type III secretion system protein SpaS [*Yersinia nurmii*]<br>Length: 350<br>97/98(350) 1–350	WP_049596339.1

PL78_18170 (164)<br>SicA	chaperone protein SicA [*Yersinia nurmii*]<br>Length: 164<br>98/100(164) 1–164	WP_049596338.1
	chaperone protein SicA [*Serratia fonticola*]<br>Length: 158<br>71/90(151) 1–151	WP_037412608.1
PL78_18175 (630)<br>SipB	hypothetical protein [*Yersinia nurmii*]<br>Length: 630<br>95/98(630) 1–630	WP_049596337.1
	hypothetical protein [*Serratia fonticola*]<br>Length: 609<br>47/67(604) 12–605	WP_046808152.1
PL78_18180 (400)<br>SipC	hypothetical protein [*Yersinia nurmii*]<br>Length: 400<br>96/97(399)1–399	WP_049596336.1
	hypothetical protein [*Serratia fonticola*]<br>Length: 364<br>33/51(281) 47–323	WP_024485265.1
PL78_18185 (408)<br>SipD	cell invasion protein SipD, partial [*Chromobacterium violaceum*]<br>Length: 283<br>47/66(212)74–281	WP_045050982.1
	hypothetical protein [*Yersinia nurmii*]<br>Length: 407<br>89/92(409) 1–407	WP_049596335.1
PL78_18190 (752)<br>SipA	hypothetical protein [*Yersinia nurmii*]<br>Length: 805<br>86/91(444) 1–443	WP_049596334.1
	hypothetical protein [*Serratia fonticola*]<br>Length: 733<br>28/48(194) 60–247	WP_024485266.1

PL78_18195(85)	hypothetical protein [*Yersinia nurmii*]<br>Length: 77<br>92/97(77) 1–77	WP_049596333.1
	acyl carrier protein [Candidatus Regiella insecticola]<br>Length: 91<br>43/69(75) 2–76	WP_039908335.1
PL78_18205 (402)<br>PrgH	type III secretion system needle complex protein PrgH [*Yersinia nurmii*]<br>Length: 402<br>96/98(402) 1–402	embCND83112.1
	hypothetical protein [*Serratia fonticola*]<br>Length: 401<br>46/66(401) 2–401	WP_046808154.1
PL78_18210 (87)<br>PrgI	type III secretion system needle complex protein PrgI [*Yersinia nurmii*]<br>Length: 87<br>92/95(87) 1–87	WP_049596331.1
	type III secretion apparatus needle protein [*Cedecea davisae* DSM 4568]<br>Length: 90<br>76/81(76) 15–90	gbEPF14983.1
PL78_18215 (100)<br>PrgJ	hypothetical protein [*Yersinia nurmii*]<br>Length: 100<br>97/98(100)1–100	WP_049596330.1
	hypothetical protein [*Serratia fonticola*]<br>Length: 97<br>53/80(96) 2–97	WP_024485270.1
PL78_18220(244)<br>PrgK	pathogenicity island 1 effector protein [*Yersinia nurmii*]<br>Length: 244<br>98/98(244) 1–244	WP_049596329.1
	type III secretion apparatus lipoprotein [*Serratia fonticola*]<br>Length: 235<br>66/83(229) 2–230	WP_024485271.1
PL78_18225 (190)<br>OrgA	hypothetical protein [*Yersinia nurmii*]<br>Length: 190<br>94/97(190)1–190	WP_049596328.1
	hypothetical protein [*Serratia fonticola*]<br>Length: 199<br>46/63(182) 15–196	WP_024485272.1

PL78_18230 (233)<br>OrgB	hypothetical protein [*Yersinia nurmii*]<br>Length: 233<br>95/98(233) 1–233	WP_049596327.1
	hypothetical protein [*Serratia fonticola*]<br>Length: 230<br>42/65(149) 1–226	WP_024485273.1
PL78_18235 (73)	hypothetical protein [*Yersinia nurmii*]<br>Length: 70<br>97/99(58) 1–70<br>Sequence ID: emb|CND82903.1| Length: 70<br>81/90(70) 1–70	WP_049596326.1
	hypothetical protein [*Serratia fonticola*]<br>Length: 71<br>48/68(58) 14–71	WP_024485274.1
PL78_18240 (197)	Uncharacterised protein [*Yersinia nurmii*]<br>Length: 196<br>91/94(196) 1–196	embCND82874.1
	hypothetical protein [*Serratia fonticola*]<br>Length: 197<br>44/61(201) 1–196	WP_024485275.1
PL78_18245 (438)	oxidoreductase [*Yersinia nurmii*]<br>Length: 438<br>97/98(438) 1–438	WP_049596324.1
	oxidoreductase [*Serratia fonticola*]<br>Length: 438<br>80/90(438) 1–438	WP_046808249.1
PL78_18250 (367)	agmatine deiminase [*Yersinia nurmii*]<br>Length: 367<br>91/95(367) 1–367	WP_049596323.1
	agmatine deiminase [*Marinomonas mediterranea*]<br>Length: 367<br>67/78(366) 1–365	WP_013660875.1

Yersinia entomophaga T3SSYE2.

**Locus (Amino Acid Residues)**	**Closest BlastP Orthologue [Species]**<br>**Length: Amino Acid Length of Orthologue**<br>**% Identity/% similarity (over the Indicated Range of Amino Acid Residues) in Relation to Target Sequence.**<br>**Protein Domain**	**Accession Number**
PL78_14480 (851)	DNA mismatch repair protein MutS [*Yersinia nurmii*]<br>Length: 851<br>99/99(851) 1–851	WP_049601201.1
PL78_14490 (147)	membrane protein [*Yersinia nurmii*]<br>Length: 147<br>90/93(147) 1–147	embCNF08019.1
	hypothetical protein BD65_2656 [*Yersinia ruckeri*]<br>Length: 149<br>63/75(149) 1–149	gbAJI94952.1
PL78_14495 (120)	lytic transglycosylase [*Yersinia nurmii*]<br>Length: 159<br>81/91(159) 1–159	WP_049601195.1
	lytic transglycosylase [*Yersinia ruckeri*]<br>Length: 158<br>61/77(145) 11–155	WP_038244579.1
PL78_14500 (117)	helicase [*Halomonas* sp. TG39a]<br>Length: 907<br>31/47(122) 82–199	WP_035576868.1
	Uncharacterised protein [*Yersinia nurmii*]<br>Length: 138<br>73/83(138) 1–138	embCNF07957.1
PL78_14505 (224)<br>OrgB	invasion protein OrgB [*Yersinia nurmii*]<br>Length: 224<br>82/90(224) 1–224	embCNF07929.1
	hypothetical protein [*Yersinia ruckeri*]<br>Length: 222<br>40/60(213) 10–222	WP_042527542.1

PL78_14510 (181)	hypothetical protein [*Yersinia nurmii*]<br>Length: 181<br>84/91(160) 21–180	WP_049601187.1
	hypothetical protein [*Yersinia ruckeri*]<br>Length: 176<br>44/67(158) 17–174	WP_038251258.1
PL78_14515 (248)<br>Prgk	type III secretion protein [*Yersinia nurmii*]<br>Length: 247<br>91/94(247) 1–247	WP_049601185.1
	type III secretion protein [*Yersinia ruckeri*]<br>Length: 252<br>69/80(247) 1–243	WP_042527545.1
PL78_14520 (101)<br>PrgJ	hypothetical protein [*Yersinia ruckeri*]<br>Length: 100<br>44/61(101) 1–100	WP_042527547.1
	hypothetical protein [*Yersinia nurmii*]<br>Length: 101<br>94/97(101) 1–101	WP_049601183.1
PL78_14525 (100)<br>PrgI	hypothetical protein [*Yersinia nurmii*]<br>Length: 99<br>36/51(97) 1–99	WP_049601181.1
	hypothetical protein [*Yersinia ruckeri*]<br>Length: 104<br>36/51(97) 8–103	WP_042527549.1
PL78_14530 (422)<br>PrgH	type III secretion system needle complex protein PrgH [*Yersinia nurmii*]<br>Length: 422<br>85/92(422) 1–422	embCNF07697.1
	hypothetical protein [*Yersinia ruckeri*]<br>Length: 395<br>39/59(411) 3–395	WP_042527551.1

PL78_14535 (150)<br>AraC	hypothetical protein [*Yersinia ruckeri*]<br>Length: 264<br>42/57(143) 14–156	WP_052470857.1
	hypothetical protein [*Yersinia nurmii*]<br>Length: 261<br>83/89(131) 7–137	WP_049601175.1
PL78_14540 (567)<br>InvG<br>Ysac	type III secretion system protein [*Yersinia nurmii*]<br>Length: 567<br>95/97(567) 1–567	WP_049601173.1
	type III secretion system protein [*Yersinia ruckeri*]<br>Length: 570<br>74/86(565) 5–569	WP_042527553.1
PL78_14545 (366)<br>InvE<br>Ysaw	hypothetical protein [*Yersinia nurmii*]<br>Length: 366<br>90/95(366) 1–366	WP_049601168.1
	hypothetical protein [*Yersinia ruckeri*]<br>Length: 368<br>67/82(357) 1–357	WP_038244567.1
PL78_14550 (691)<br>InvA	type III secretion system protein InvA [*Yersinia ruckeri*]<br>Length: 689<br>80/88(689) 1–689	WP_045844472.1
	type III secretion system protein InvA [*Yersinia nurmii*]<br>Length: 689<br>95/97(689) 1–689	WP_049601166.1
PL78_14555 (140)<br>InvB<br>Ysak	type III secretion system protein [*Yersinia nurmii*]<br>Length: 135<br>91/95(135) 1–135	WP_049601164.1
	type III secretion system protein [*Yersinia ruckeri*]<br>Length: 135<br>66/78(134) 1–134	WP_042527558.1

PL78_14560 (430)<br>SpaL<br>InvC	ATP synthase SpaL [*Yersinia nurmii*]<br>Length: 399<br>92/96(399) 1–399	embCNF07457.1
	ATP synthase [*Yersinia nurmii*]<br>Length: 430<br>92/96(430) 1–430	WP_049601162.1
PL78_14565 (153)	hypothetical protein [*Yersinia nurmii*]<br>Length: 153<br>82/90(153) 1–153	WP_049601160.1
	hypothetical protein [*Yersinia ruckeri*]<br>Length: 159<br>49/70(152) 1–152	WP_038244563.1
PL78_14570 (365)	hypothetical protein [*Yersinia ruckeri*]<br>Length: 411<br>53/73(75) 335–409	WP_042527563.1
	hypothetical protein [*Yersinia nurmii*]<br>Length: 365<br>75/82(365) 1–365	WP_049601159.1
PL78_14575 (310)<br>SpoA<br>Ysaq	hypothetical protein [*Yersinia nurmii*]<br>Length: 310<br>84/92(310) 1–310	WP_049601155.1
	hypothetical protein [*Yersinia ruckeri*]<br>Length: 308<br>51/67(310) 1–308	WP_038244561.1
PL78_14580 (218)<br>Spap<br>YsaR	type III secretion system protein [*Yersinia nurmii*]<br>Length: 218<br>96/98(218) 1–218	WP_049601152.1
	type III secretion system protein [*Yersinia ruckeri*]<br>Length: 218<br>83/92(218) 1–218	WP_042527567.1

PL78_14585 (87)<br>Spaq	type III secretion apparatus protein [*Yersinia nurmii*]<br>Length: 87<br>94/97(87) 1–87	WP_049601148.1
	hypothetical protein [*Yersinia ruckeri*]<br>Length: 87<br>88/97(86) 1–86	WP_004722103.1
PL78_14590 (264)<br>SpaR<br>YscT	type III secretion system protein [*Yersinia nurmii*]<br>Length: 264<br>94/96(264) 1–264	WP_049601145.1
	type III secretion system protein [*Yersinia ruckeri*]<br>Length: 264<br>79/90(264) 1–264	WP_042527569.1
PL78_14595 (376)<br>SpaS	type III secretion system protein [*Yersinia ruckeri*]<br>Length: 386<br>76/89(377) 1–374	WP_042527571.1
	type III secretion system protein [*Yersinia nurmii*]<br>Length: 376<br>94/98(376) 1–376	WP_049601142.1
PL78_14600 (170)<br>SicA	type III secretion protein [*Yersinia ruckeri*]<br>Length: 170<br>84/91(167) 1–167	gbKFE37947.1
	type III secretion protein [*Yersinia nurmii*]<br>Length: 170<br>96/97(170) 1–170	WP_049601141.1
PL78_14605 (643)<br>SipB<br>Ysp1	hypothetical protein [*Yersinia nurmii*]<br>Length: 643<br>89/93(643) 1–643	WP_049601136.1
	hypothetical protein [*Yersinia ruckeri*]<br>Length: 659<br>43/64(676) 1–643	WP_042527573.1

PL78_14610 (410)<br>SipC<br>Yspc	hypothetical protein [*Yersinia nurmii*]<br>Length: 410<br>84/91(410) 1–410	WP_049601135.1
	hypothetical protein [*Yersinia ruckeri*]<br>Length: 503<br>30/54(337) 179–503	WP_038275434.1
PL78_14615 (682)<br>SipD	hypothetical protein [*Yersinia nurmii*]<br>Length: 341<br>86/92(341) 1–341	WP_049601131.1
	hypothetical protein [*Yersinia ruckeri*]<br>Length: 365<br>44/64(226) 143–365	WP_038275436.1
PL78_14620 (93)	acyl carrier protein [*Yersinia nurmii*]<br>Length: 91<br>84/90(92) 1–89	embCNF06932.1
	hypothetical protein [*Yersinia ruckeri*]<br>Length: 94<br>55/75(73) 19–91	WP_052470858.1
PL78_14625 (128)	secretion protein [*Yersinia nurmii*]<br>Length: 128<br>93/95(128) 1–128	WP_049601130.1
	secretion protein [*Yersinia mollaretii*]<br>Length: 128<br>54/72(128) 1–128	WP_004874038.1
PL78_14630 (547)	fumarate hydratase [*Yersinia ruckeri*]<br>Length: 547<br>98/98(547) 1–547	WP_004722663.1
	fumarate hydratase [*Yersinia nurmii*]<br>Length: 547<br>99/99(547) 1–547	WP_049601127.1

Yersinia entomophaga general secretion pathway protein T2SS.

**Locus (Amino Acid Residues)**	**Closest BlastP Orthologue [Species]**<br>**Length: Amino Acid Length of Orthologue**<br>**% Identity/% Similarity (over the Indicated Range of Amino Acid Residues) in Relation to Target Sequence.**<br>**Protein Domain**	**Accession Number**
PL78_08915 (222)<br>LysR	transcriptional regulator [*Yersinia nurmii*]<br>Length: 222<br>93/95(222) 1–222	WP_049599794.1
	hypothetical protein [*Yersinia ruckeri*]<br>Length: 222<br>59/76(222) 1–222	WP_042525802.1
PL78_08920 (98)	transposase [*Yersinia enterocolitica*]<br>Length: 180<br>74/75(97) 22–118	WP_050927038.1
	transposase [Serratia sp. Leaf51]<br>Length: 180<br>74/75(96) 22–117	WP_056779158.1
PL78_08925 (65)	transposase [Pluralibacter gergoviae]<br>Length: 88<br>82/88(60) 1–60	WP_048282832.1
	transposase [*Yersinia enterocolitica*]<br>Length: 88<br>82/88(60) 1–60	WP_050927036.1
PL78_08930 (280)<br>GspK	general secretion pathway protein K [*Yersinia nurmii*]<br>Length: 259<br>78/87(256) 1–256	embCNE84927.1
	hypothetical protein [*Yersinia ruckeri*]<br>Length: 321<br>33/52(315) 5–317	WP_038276190.1

PL78_08935 (204)<br>GspJ	type II secretion system protein GspJ [*Yersinia nurmii*]<br>Length: 213<br>87/94(204) 10–213	WP_049599786.1
	general secretion pathway protein J [*Serratia marcescens* FGI94]<br>Length: 202<br>34/56(198) 11–200	gbAGB84035.1
PL78_08940 (127)<br>GspI	type II secretion system protein GspI [*Yersinia nurmii*]<br>Length: 127<br>82/87(127) 1–127	WP_049599784.1
	general secretion pathway protein I [Aeromonas diversa CDC 2478–85]<br>Length: 119<br>32/61(108) 2–104	gbENY71267.1
PL78_08945 (185)	hypothetical protein [*Yersinia nurmii*]<br>Length: 183<br>81/90(182) 1–182	WP_049599781.1
	hypothetical protein [*Yersinia ruckeri*]<br>Length: 192<br>29/48(173) 6–171	WP_038241044.1
PL78_08950 (102)<br>GspI	type II secretion system protein GspG [*Yersinia nurmii*]<br>Length: 150<br>93/96(150) 1–150	WP_049599778.1
	type II secretion system protein GspG [*Yersinia ruckeri*]<br>Length: 149<br>76/85(143) 1–143	WP_038241042.1
PL78_08955 (399)	hypothetical protein [*Yersinia nurmii*]<br>Length: 399<br>93/97(399) 1–399	WP_049599774.1
	hypothetical protein [*Yersinia ruckeri*]<br>Length: 395<br>50/71(397) 1–393	WP_042525816.1

PL78_08960 (495)<br>GspE	type II secretion system protein GspE [*Yersinia nurmii*]<br>Length: 502<br>96/98(495) 8–502	WP_049599771.1
	type II secretion system protein E [*Yersinia ruckeri* ATCC 29473]<br>Length: 491<br>71/85(491) 1–491	gbKGA50644.1
PL78_08965 (651)<br>GspD	general secretion pathway protein GspD [*Yersinia nurmii*]<br>Length: 680<br>94/96(645) 29–673	WP_053215274.1
	type II secretion system protein GspD [*Yersinia ruckeri*]<br>Length: 650<br>68/83(633) 21–650	WP_038241119.1
PL78_08970 (187)<br>GspC	hypothetical protein [*Yersinia nurmii*]<br>Length: 189<br>79/90(188) 1–188	WP_049599768.1
	General secretion pathway protein C [*Yersinia ruckeri* ATCC 29473]<br>Length: 188<br>32/50(186)	gbEEP93904.1
PL78_08975 (139)	hypothetical protein [*Yersinia nurmii*]<br>Length: 139<br>100/100(139) 1–139	WP_049599767.1
	hypothetical protein yruck0001_4150 [*Yersinia ruckeri* ATCC 29473]<br>Length: 157<br>194/97(139) 19–157	gbEEP99541.1
PL78_08980 (94)	No significant similarity	
PL78_08985<br>tRNA–Gly		

Table S4. *Yersinia entomophaga* accessory virulence determinants: Repeats in Toxin cluster, CdtAB, VIP2, adenylate cyclase, chitinase and chitin binding protein associated regions and putative hemolysins. For graphic depiction refer to Figure 5 of the associated manuscript.

Yersinia entomophaga Repeats in Toxin associated cluster.

Locus (Amino Acid Residues)	Closest BlastP Orthologue [Species]<br>Length: Amino Acid Length of Orthologue<br>% Identity/% Similarity (over the Indicated Range of Amino Acid Residues) in Relation to Target Sequence.<br>Protein Domain	Accession Number
PL78_16895 (216)	transcriptional regulator [*Yersinia nurmii*]<br>Length: 216<br>99/100(216) 1–216	WP_049597066.1
	transcriptional regulator [*Yersinia ruckeri*]<br>Length: 216<br>99/100(216) 1–216	WP_042525316.1
PL78_16900 (907)	tRNA(5–methylaminomethyl–2–thiouridylate) methyltransferase [*Yersinia nurmii*]<br>Length: 907<br>96/98(907) 1–907	WP_049597067.1
	tRNA(5–methylaminomethyl–2–thiouridylate) methyltransferase [*Yersinia ruckeri*]<br>Length: 907<br>85/91(907) 1–907	WP_042525315.1
PL78_16905 (384)<br>MFS transporter	MFS transporter [*Yersinia nurmii*]<br>Length: 384<br>98/99(384)1–384	WP_049597068.1
	MFS transporter [*Yersinia ruckeri*]<br>Length: 384<br>90/95(384) 1–384	WP_042528242.1
PL78_16910 (4660)<br>RTX	autotransporter adhesin [*Yersinia nurmii*]<br>Length: 4660<br>93/95(4661) 1–4659	WP_049597069.1
	adhesin [*Yersinia enterocolitica*]<br>Length: 4221<br>58/72(4103) 179–4220	WP_050915363.1

PL78_16915 (461)	outer membrane channel protein [*Yersinia nurmii*]<br>Length: 461<br>98/99(461) 1–461	embCNE14355.1
	MULTISPECIES: type I secretion system outer membrane protein [Yersinia]<br>Length: 461<br>85/91(463) 1–461	WP_050142178.1
PL78_16920 (713)<br>T1SS (CyaE)	putative toxin transport protein [*Yersinia nurmii*]<br>Length: 713<br>97/98(713) 1–713	embCNE14391.1
	putative toxin transport protein [*Yersinia massiliensis*]<br>Length: 760<br>87/92(712) 51–760	embCNH58774.1
PL78_16925 (389)<br>HlyD	secretion protein HlyD [*Yersinia nurmii*]<br>Length: 389<br>99/99(389) 1–289	WP_049597070.1
	secretion protein HlyD [*Yersinia frederikseniir*]<br>Length: 389<br>88/95(389) 1–389	WP_050140418.1
PL78_16930 (370)	membrane protein [*Yersinia nurmii*]<br>Length: 370<br>97/98(370)1–370	WP_049597071.1
	membrane protein [*Yersinia ruckeri*]<br>Length: 376<br>90/94(376)1–376	WP_038251489.1
PL78_16945 (183)	GNAT family acetyltransferase [*Yersinia nurmii*]<br>Length: 183<br>97/98(183) 1–183	WP_049597072.1
	GNAT family acetyltransferase [*Yersinia ruckeri*]<br>Length: 178<br>81/88(178) 1–178	WP_042525312.1

PL78_16950 (376)	beta–lactam binding protein AmpH [*Yersinia nurmii*]<br>Length: 376<br>99/98(367)10–376	embCNE14590.1
	beta–lactam–binding protein [*Yersinia ruckeri*]<br>Length: 381<br>91/94(367)15–381	WP_042525310.1
PL78_16955 (475)	hypothetical protein yinte0001_11730 [*Yersinia intermedia* ATCC 29909]<br>Length: 512<br>87/91(485) 5–489	gbEEQ17977.1
	MULTISPECIES: MFS transporter [Yersinia]<br>Length: 508<br>87/91(485) 1–485	WP_032907368.1
PL78_16960 (330)	MBL fold hydrolase [*Yersinia nurmii*]<br>Length: 330<br>94/96(330) 1–330	WP_045844204.1
	MBL fold metallo–hydrolase [*Yersinia ruckeri*]<br>Length: 330<br>83/89(330) 1–330	WP_049597075.1
PL78_16965 (325)	hypothetical protein [*Yersinia nurmii*]<br>Length: 325<br>98/98(325) 1–325	WP_050475862.1
	hypothetical protein [Herbaspirillum rhizosphaerae]<br>Length: 326<br>71/82(324) 1–324	WP_049597076.1

Yersinia entomophaga putative virulence cluster CdtAB.

Locus (Amino Acid Residues)	Closest BlastP orthologue [Species]<br>Length: Amino Acid Length of Orthologue<br>% Identity/% Similarity (over The Indicated Range of Amino Acid Residues) in Relation to Target Sequence.<br>Protein Domain	Accession Number
PL78_18410	hypothetical protein [*Yersinia nurmii*]<br>Length: 241<br>96/97(241) 1–241	WP_049600373.1
	hypothetical protein [*Serratia fonticola*]<br>Length: 249<br>46/63(241) 12–249	WP_059200486.1
PL78_18415 (371)	D–alanine—D–alanine ligase [*Yersinia nurmii*]<br>Length: 371<br>98/99(371) 1–367	WP_049600376.1
	D–alanine—D–alanine ligase [*Yersinia ruckeri*]<br>Length: 368<br>92/97(367) 1–367	WP_038276041.1
PL78_18420 (211)	yheo–like PAS domain [*Yersinia nurmii*]<br>Length: 220<br>95/97(220) 1–220	embCNE94733.1
	yheo–like PAS domain [*Yersinia frederikseniir*]<br>Length: 230<br>6/92(217) 12–228	embCNK81303.1
PL78_18425 (323)	serine dehydratase [*Yersinia nurmii*]<br>Length: 323<br>97/98(323) 1–323	WP_049600379.1
	serine dehydratase [*Yersinia intermedia*]<br>Length: 322<br>79/88(321) 1–321	WP_050077290.1

PL78_18430 (293)	alpha/beta hydrolase [*Yersinia nurmii*]<br>Length: 293<br>95/96(293) 1–293	WP_049600386.1
	alpha/beta hydrolase [*Serratia marcescens*]<br>Length: 294<br>63/77(294) 1–294<br>pfam00561; COG1075	WP_033635162.1
PL78_18435 (108)	acid–resistance protein [*Yersinia nurmii*]<br>Length: 108<br>95/98(108) 1–108	WP_049600389.1
	acid–resistance protein [*Yersinia rohdei*]<br>Length: 110<br>76/86(110) 1–110	WP_032816440.1
PL78_18440 (190)<br>CdtA	hypothetical protein [*Yersinia nurmii*]<br>Length: 190<br>83/90(189) 1–189	WP_049600392.1
	hypothetical protein [*Yersinia ruckeri*]<br>Length: 191<br>59/74(182) 10–189<br>pfam03498: CDtoxinA	WP_052470825.1
PL78_18445 (296)<br>CdtB	cytolethal distending toxin subunit B [*Yersinia ruckeri*]<br>Length: 298<br>64/78(294)1–294	embCNB14664.1
	hypothetical protein [*Yersinia nurmii*]<br>Length: 296<br>91/96(296) 1–296	WP_049600395.1
PL78_18450 (466)	hypothetical protein [*Yersinia nurmii*]<br>Length: 466<br>94/97(466) 1–466	WP_049600397.1
	hypothetical protein [*Yersinia ruckeri*]<br>Length: 463<br>73/85(466) 1–463	WP_049689405.1

PL78_18455 (282)	kinase [*Yersinia nurmii*]<br>Length: 282<br>99/99(282) 1–282	WP_049600400.1
	serine/threonine protein kinase [*Yersinia ruckeri*]<br>Length: 282<br>93/96(282) 1–282	WP_004721381.1

Yersinia entomophaga putative VIP2 associated region.

Locus (Amino Acid Residues)	Closest BlastP Orthologue [Species]<br>Length: Amino Acid Length of Orthologue<br>% Identity/% Similarity (over the Indicated Range of Amino Acid Residues) in Relation to Target Sequence.<br>Protein Domain	Accession Number
PL78_16130 (293)	transcriptional regulator [*Yersinia nurmii*]<br>Length: 293<br>96/96(293) 1–293	WP_049596942.1
	transcriptional regulator [*Yersinia ruckeri*]<br>Length: 287<br>86/92(287) 1–287	WP_042525447.1
PL78_16135 (158)	Na(+)–translocating NADH–quinone reductase subunit E [*Yersinia nurmii*]<br>Length: 158<br>99/99(158) 1–158	WP_049596943.1
	a+–translocating NADH–quinone reductase subunit E [*Yersinia ruckeri*]<br>Length: 158<br>91/93(158) 1–158	WP_004717931.1
PL78_16140 (321)	glucokinase [*Yersinia nurmii*]<br>Length: 321<br>96/97(321) 1–321	WP_049596944.1
	glucokinase [*Yersinia ruckeri*]<br>Length: 321<br>90/94(321) 1–321	WP_042525445.1
PL78_16145 (402)<br>VIPA	hypothetical protein [*Yersinia enterocolitica*]<br>Length: 400<br>50–67(403) 1–400	WP_005175826.1
	NAD:arginine ADP–ribosyltransferase [*Yersinia enterocolitica*]<br>Length: 402<br>50/66(399) 9–402<br>(pfam01129)	embCRY18359.1

PL78_16150 (414)	membrane protein [*Yersinia nurmii*]<br>Length: 414<br>98/99(414) 1–414	WP_049596945.1
	membrane protein [*Yersinia ruckeri*]<br>Length 415<br>92/95(415) 1–415	WP_004717929.1

Yersinia entomophaga putative virulence cluster adenylate cyclase associated region.

**Locus (Amino Acid Residues)**	**Closest BlastP Orthologue [Species]**<br>**Length: Amino Acid Length of Orthologue**<br>**% Identity/% Similarity (over the Indicated Range of Amino Acid Residues) in Relation to Target Sequence.**<br>**Protein Domain**	**Accession number**
PL78_08385 (330)	serine/threonine dehydratase [*Yersinia nurmii*]<br>Length: 330<br>99/100(330) 1–330	WP_049599166.1
	serine/threonine dehydratase [*Yersinia aldovae*]<br>ength: 330<br>94/97(330) 1–330	WP_004699735.1
PL78_08390 (61)	Nil similarity	
PL78_08395 (351)	toxin protein [*Yersinia nurmii*]<br>Length: 402<br>89/95(350)52–401	embCNE75600.1
	adenylate cyclase [Serratia sp. Leaf50]<br>Length: 369<br>56/76(356) 13–402	WP_055772549.1
	adenylate cyclase–1 [Candidatus Hamiltonella defensa]<br>Length: 437<br>55/74(345) 77–415	WP_015873608.1
	calmodulin–sensitive adenylate cyclase [Bacillus anthracis]<br>Length: 800<br>35/52(201) 306–503<br>pfam03497 Anthrax_toxA	WP_000197748.1

PL78_08400 (931)	peptidase M16 [*Yersinia nurmii*]<br>Length: 931<br>94/96(931)1–931	WP_049599163.1
	peptidase M16 [*Yersinia enterocolitica*]<br>Length: 927<br>77/87(931) 1–927	WP_050157122.1

Yersinia entomophaga chitinase.

Locus (Amino Acid Residues)	Closest BlastP Orthologue [Species]<br>Length: Amino Acid Length of Orthologue<br>% Identity/% Similarity (over the Indicated Range of Amino Acid Residues) in Relation to Target Sequence.<br>Protein Domain	Accession Number
PL78_11905 (450)	glutathione–disulfide reductase [*Yersinia nurmii*]<br>Length: 450<br>99/99(450) 1–450	WP_049597320.1
	glutathione–disulfide reductase [*Yersinia intermedia*]<br>Length: 450<br>97/98(450) 1–450	WP_050073664.1
PL78_11910 (351)	glycosyl hydrolase family protein [*Yersinia nurmii*]<br>Length: 352<br>98/98(352)1–352	embCNE26027.1
	chitinase [Paenibacillus dauci]<br>Length: 414<br>60/77(341) 13–351	WP_052714100.1
PL78_11915 (396)	cystathionine beta–lyase [*Yersinia nurmii*]<br>Length: 396<br>97/98(396) 1–396	WP_049597321.1
	cystathionine beta–lyase [*Yersinia ruckeri*]<br>Length: 396<br>96/98(396) 1–396	WP_038242896.1

Yersinia entomophaga chitin binding fimbrial cluster, Chi–Fim.

Locus (Amino Acid Residues)	Closest BlastP Orthologue [Species]<br>Length: Amino Acid Length of Orthologue<br>% Identity/% Similarity (over the Indicated Range Of Amino Acid Residues) in Relation to Target Sequence.<br>Protein Domain	Accession Number
PL78_08260 (176)	outer membrane lipoprotein Blc [*Yersinia nurmii*]<br>Length: 176<br>98/99(176) 1–176	embCNE76662.1
	membrane protein [*Yersinia ruckeri*]<br>Length: 180<br>89/96(174) 1–174	WP_042528366.1
PL78_08265 (104)	multidrug transporter [*Yersinia nurmii*]<br>99/100(104) 1–104	WP_049599231.1
	multidrug transporter [*Yersinia ruckeri*]<br>95/99(104) 1–104	WP_038241885.1
PL78_08270 (174)	fimbrial protein [*Yersinia nurmii*]<br>Length: 174<br>99/99(174) 1–174	WP_049599227.1
	fimbrial protein [*Yersinia ruckeri*]<br>Length: 174<br>89/95(174) 1–174	WP_042527048.1
PL78_08275 (660)	pilus assembly protein [*Yersinia nurmii*]<br>Length: 805<br>99/99(660) 128–787	WP_049599224.1
	pilus assembly protein [*Yersinia ruckeri*]<br>Length: 803<br>85/92(659) 126–784	WP_052470850.1

PL78_08280(244)	fimbrial protein [*Yersinia nurmii*] Length: 244<br>Legth: 244<br>97/97(244) 1–244	WP_049599223.1
	fimbrial protein [*Yersinia ruckeri*]<br>Length: 244<br>81/89(244) 1–244	WP_042527047.1
PL78_08285 (330)<br>pfam00419	hypothetical protein [*Yersinia nurmii*]<br>Length: 330<br>97/98(330) 1–330	WP_049599220.1
	fimbrial family protein [*Yersinia ruckeri*]<br>Length: 330<br>88/93(330) 1–330	gbAJI95306.1
PL78_08290 (203)	helix–turn–helix transcriptional regulator [*Yersinia ruckeri*]<br>97/98(202) 1–202	WP_038241888.1
PL78_08295 (197)	chitin–binding protein [*Yersinia ruckeri*]<br>Length: 197<br>89/93(196) 1–196	WP_052470849.1
PL78_08300 (160)	elongation factor P [*Yersinia ruckeri*]<br>Length: 188<br>100/100(160) 1–160	WP_004722438.1
	elongation factor P [*Yersinia nurmii*]<br>Length: 188<br>99/99(160) 1–160	WP_049599212.1

Yersinia entomophaga putative chitin binding fimbrial cluster, Chi–SrfAB.

Locus (Amino Acid Residues)	Closest BlastP Orthologue [Species]<br>Length: Amino Acid Length of Orthologue<br>% Identity/% Similarity (over the Indicated Range of Amino Acid Residues) in Relation To Target Sequence.<br>Protein Domain	Accession Number
PL78_05305 (216)	Vat family streptogramin A O–acetyltransferase [*Yersinia nurmii*]<br>Length: 216<br>95/96(216) 1–216	WP_049598340.1
	streptogramin A acetyl transferase [Enterobacter sp. 638]<br>Length: 216<br>74/90(210) 1–216	gbABP59079.1
PL78_05310 (216)	chitin–binding protein [*Yersinia nurmii*]<br>Length: 216<br>93/95(216)–1–216	WP_049598342.1
	chitin–binding protein [*Yersinia intermedia*]<br>Length: 216<br>71/78(215)–1–215	WP_005183097.1
PL78_05315 (464)<br>SrfA	virulence factor [*Yersinia nurmii*]<br>Length: 444<br>91/92(451)1–444	embCNE60077.1
	virulence effector protein [*Yersinia ruckeri*]<br>Length: 442<br>65/71(471)1–442	WP_004717438.1
PL78_05320 (994)<br>SrfB	virulence factor SrfB [*Yersinia nurmii*]<br>Length: 994<br>98/98(994) 1–994	WP_049598346.1
	virulence factor SrfB [*Yersinia ruckeri*]<br>Length 994<br>87/93(994) 1–994	WP_038242595.1

PL78_10835 (460)<br>virulence factor	virulence factor [*Yersinia nurmii*]<br>Length: 816<br>94/96(433) 1–433	WP_049598348.1
	virulence factor [*Yersinia ruckeri*]<br>Length: 816<br>72/83(433) 1–433<br>pfam10139: Virul_Fac putative bacterial virulence factor	WP_038275949.1
PL78_05330 (189)	hypothetical protein [*Yersinia nurmii*]<br>Length 189<br>96/97(189) 1–189	WP_049598349.1
	serine hydrolase family protein [*Yersinia ruckeri*]<br>Length: 186<br>78/87(185) 1–185	gbAJI94666.1
	hypothetical protein [Xenorhabdus doucetiae]<br>Length: 185<br>87/73(186) 1–185	WP_045973197.1
PL78_05335 (195)	Uncharacterized distant relative of cell wall–associated hydrolases [*Yersinia nurmii*]<br>Length: 196<br>98/98(195) 2–196	embCNE60215.1
	hypothetical protein MU9_2339 [Morganella morganii subsp. morganii KT]<br>63/79(188) 9–196	gbAGG31384.1

Yersinia entomophaga putative hemolysin's.

**Locus (Amino Acid Residues)**	**Closest BlastP Orthologue [Species]**<br>**Length: Amino Acid Length of Orthologue**<br>**% identity/% Similarity (over the Indicated Range of Amino Acid Residues) in Relation to Target Sequence.**<br>**Protein Domain**	**Accession Number**
PL78_04365 (1624)	hemolysin [*Yersinia nurmii*]<br>Length: 1625<br>96/97(1625)1–1625	WP_049597670.1
	hemolysin [*Yersinia ruckeri*]<br>Length: 1630<br>57/93 (1630)1–1628	WP_045844326.1
	hemolysin [Serratia proteamaculans]<br>Length: 1615<br>57/71(1646) 1–1615	WP_012147176.1
PL78_11060 (1645)	hemolysin [*Yersinia nurmii*]<br>Length: 1645<br>95/97(1645)1–1645	WP_049598079.1
	hemolysin [*Yersinia ruckeri*]<br>Length: 1630<br>70/82(1637)1–1627	WP_004719456.1
	hemolysin [Serratia liquefaciens]<br>Length: 1615<br>53/69(1–1615) 1–1615	WP_046372512.1
PL78_09050(221)	hemolysin III family protein [*Yersinia nurmii*]<br>Length: 221<br>98/100(221) 1–221	WP_049600002.1
	hemolysin III [*Yersinia intermedia*]<br>Length: 237<br>83/89(237) 1–237	embCQJ63471.1

Table S5. *Yersinia entomophaga* cell adhesins, fimbriae, Lipopolysaccharide and cellulose encoding clusters. For graphic depiction refer to Figure 7 of the associated manuscript.

Yersinia entomophaga fimbrial associated region.

Locus (Amino Acid Residues)	Closest BlastP Orthologue [Species]<br>Length: Amino Acid Length of Orthologue<br>% identity/% Similarity (over the Indicated Range of Amino Acid Residues) in Relation to Target Sequence.<br>Protein Domain	Accession Number
PL78_08255 (118)	fumarate reductase [*Yersinia nurmii*]<br>Length: 118<br>99/100(118) 1–118	WP_049599234.1
	fumarate reductase subunit D [*Yersinia ruckeri*]<br>97/100(118) 1–118	WP_004722457.1
PL78_08260 (176)	outer membrane lipoprotein Blc [*Yersinia nurmii*]<br>Length: 176<br>98/99(176) 1–176	embCNE76662.1
	membrane protein [*Yersinia ruckeri*]<br>Length: 180<br>89/96(174) 1–174	WP_042528366.1
PL78_08265 (104)	multidrug transporter [*Yersinia nurmii*]<br>Length: 104<br>99/100(104) 1–104	WP_049599231.1
	multidrug transporter [*Yersinia ruckeri*]<br>Length: 104<br>95/99(104) 1–104	WP_038241885.1
PL78_08270 (174)	fimbrial protein [*Yersinia nurmii*]<br>Length: 174<br>99/99(174) 1–174	WP_049599227.1
	fimbrial protein [*Yersinia ruckeri*]<br>Length: 174<br>90/95(174) 1–174	WP_042527048.1

PL78_08275 (678) FimD/PapC	pilus assembly protein [*Yersinia nurmii*]<br>Length: 805<br>98/99(678) 128–805	WP_049599224.1
	pilus assembly protein [*Yersinia ruckeri*]<br>Length: 803<br>84/91(678) 126–803	WP_052470850.1
PL78_08280 (244) PapD	fimbrial protein [*Yersinia nurmii*]<br>Length: 244<br>97/97(244) 1–244	WP_049599223.1
	fimbrial protein [*Yersinia ruckeri*]<br>Length: 244<br>81/89(244) 1–244	WP_042527047.1
PL78_08285 (330)	hypothetical protein [*Yersinia nurmii*]<br>Length: 330<br>97/98(330) 1–330	WP_049599220.1
	fimbrial family protein [*Yersinia ruckeri*]<br>Length: 330<br>88/93(330) 1–330	gbAJI95306.1
PL78_08290 (203)	helix–turn–helix transcriptional regulator [*Yersinia nurmii*]<br>Length: 199<br>77/86(202)1–198	WP_049599217.1
	helix–turn–helix transcriptional regulator [*Yersinia ruckeri*]<br>Length: 203<br>97/98(202) 1–202	WP_038241888.1
PL78_08295 (197)	chitin–binding protein [*Yersinia ruckeri*]<br>Length: 197<br>89/93(196) 1–196	WP_052470849.1
PL78_08300 (188)	elongation factor P [*Yersinia nurmii*]<br>Length: 188<br>99/100(188) 1–188	WP_049599212.1
	elongation factor P [*Yersinia ruckeri*]<br>Length: 188<br>99/99(188) 1–188	WP_004722438.1

PL78_0805 (342)	lysine 2,3–aminomutase [*Yersinia nurmii*]<br>Length: 342<br>99/99(342) 1–342	WP_049599209.1
	EF–P beta–lysylation protein EpmB [*Yersinia ruckeri*]<br>Length: 342<br>93/95(341) 1–341	WP_042527045.1

Yersinia entomophaga fimbrial associated island FAI$_{YE96}$.

Locus (Amino Acid Residues)	Closest BlastP Orthologue [Species]<br>Length: Amino acid Length of Orthologue<br>% identity/% Similarity (over the Indicated Range of Amino Acid Residues) in Relation to Target Sequence.<br>Protein Domain	Accession Number
PL78_17260<br>tRNA–Asn	tRNA–Asn<br>aagcaaaaaacccgactaagtttccttaatcgggcttctctaatatggctcctctgactggactcgaaccagtgacatacggatt<br>aacagtccgccgttctaccgactgaactacagaggaatcgtgtgaacggggcg	
PL78_17265 (425)	Raffinose permease [*Yersinia rohdei*]<br>Length: 430<br>92/95(422) 9–430	WP_004716742.1
	galactoside permease [Serratia sp. Leaf51]<br>Length: 428<br>73/86(406) 7–412	WP_056775892.1
PL78_17270 (708)	alpha–galactosidase [*Yersinia rohdei*]<br>Length: 707<br>80/89(708) 1–707	WP_049616652.1
	alpha–galactosidase [*Serratia fonticola*]<br>Length: 708<br>75/85(708) 1–708	WP_024483295.1

PL78_17275 (336)<br>Reg	transcriptional regulator [*Yersinia rohdei*]<br>Length: 336<br>75/82(333) 1–333	WP_004716744.1
	transcriptional regulator [*Sodalis praecaptivus*]<br>Length: 336<br>64/75(331) 1–329	WP_025421693.1
	transcriptional regulator [*Serratia fonticola*]<br>Length: 335<br>63/76(326) 1–324	WP_024527340.1
PL78_17280 (236)<br>Chap	molecular chaperone [*Yersinia nurmii*]<br>Length: 236<br>93/97(236) 1–236	WP_049597187.1
	molecular chaperone [*Yersinia frederikseniir*]<br>Length: 250<br>62/74(239) 7–244	WP_050141295.1
PL78_17285 (421)<br>StbD	putative fimbrial usher protein StbD [*Yersinia nurmii*]<br>Length: 440<br>98/98(421) 20–440	embCNE17004.1
	hypothetical protein [*Yersinia enterocolitica*]<br>Length: 444<br>81/90(416) 29–444	WP_019082823.1
PL78_17290 (856)<br>Fim	fimbrial protein [*Yersinia nurmii*]<br>Length: 858<br>96/98(858) 1–858	WP_049597131.1
	fimbrial protein [*Yersinia enterocolitica*]<br>Length: 874<br>65/77(876) 1–874	WP_050160997.1
PL78_17295 (244)<br>PapD	pilus assembly protein [*Yersinia nurmii*]<br>Length: 244<br>96/96(244) 1–244	WP_049597132.1
	pilus assembly protein [*Yersinia mollaretii*]<br>Length: 243<br>73/82(244) 1–243	WP_049648727.1

PL78_17300 (176)<br>Fim	fimbrial protein [*Yersinia nurmii*]<br>Length: 176<br>98/98(176) 1–176	WP_049597133.1
	fimbrial protein [*Yersinia pekkanenii*]<br>Length: 176<br>76/88(176) 1–176	WP_049613690.1
PL78_17305 (1207)	histidine kinase [*Yersinia nurmii*]<br>Length: 1207<br>95/97(1207) 1–1207	WP_049613690.1
	putative virulence sensor protein [*Yersinia intermedia*]<br>Length: 1288<br>68/81(1234) 22–1273	embCRY76056.1
PL78_17310 (209)<br>LuxR	putative two–component response regulator [*Yersinia nurmii*]<br>Length: 209<br>98/99(209) 1–209	embCNE17197.1
	DNA–binding response regulator [*Yersinia intermedia*]<br>Length: 210<br>93/96(208) 3–210	WP_050075308.1
PL78_17315 (59)	hypothetical protein [*Yersinia nurmii*]<br>Length: 59<br>91/93(58) 1–58	WP_049597134.1
PL78_17320<br>tRNA–Asn	tRNA–Asn<br>aagcaaaaaacccgactaagtttccttaatcgggcttctctaatatggctcctctgactggactcgaaccagtgacatacggatt<br>aacagtccgccgttctaccgactgaactacagaggaatcgtgtgaacggggcg	

Yersinia entomophaga lipopolysaccharide cluster.

Locus (Amino Acid Residues)	Closest BlastP Orthologue [Species]<br>Length: Amino Acid Length of Orthologue<br>% Identity/% Similarity (over the Indicated Range of Amino Acid Residues) in Relation to Target Sequence.<br>Protein Domain	Accession Number
PL78_00680 (327)<br>DdhD	CDP–6–deoxy–delta–3,4–glucoseen reductase [*Yersinia nurmii*]<br>Length: 327<br>97/99(327) 1–327	WP_049598615.1
	CDP–6–deoxy–delta–3,4–glucoseen reductase [*Yersinia similis*]<br>Length: 329<br>83/94(329) 1–329	WP_054878293.1
PL78_00685 (257)<br>DdhA	cytidylyltransferase [*Yersinia nurmii*]<br>Length: 257<br>100/100(257) 1–257	WP_049598616.1
	glucose–1–phosphate cytidylyltransferase [*Yersinia ruckeri*]<br>Length: 257<br>99/99(257) 1–257	WP_038275604.1
PL78_00690 (299)<br>DdhB	C CDP–glucose 4%2C6–dehydratase [*Yersinia nurmii*]<br>Length: 357N<br>99/99(357) 1–357	embCNE67016.1
	CDP–glucose 4,6–dehydratase [*Yersinia pseudotuberculosis* IP 31758]<br>Length: 391<br>90/94(357)35–391	gbABS48236.1
PL78_00695 (437)<br>DdhC	lipopolysaccharide biosynthesis protein RfbH [*Yersinia nurmii*]<br>Length: 437<br>99/100(437) 1–437	WP_049598618.1
	CDP–4–keto–6–deoxy–D–glucose–3–dehydrase [*Yersinia pseudotuberculosis*]<br>Length: 437<br>97/98(437) 1–437	gbADI59424.1
PL78_00700 (283)<br>Prt	paratose synthase [*Yersinia enterocolitica*]<br>Length: 285<br>70/84(283) 1–283	WP_057647476.1

PL78_00705 (338)	CDP–paratose 2–epimerase [*Yersinia mollaretii*]<br>Length: 338<br>84/92(338)1–338	ref_049608459.1
PL78_00710 (438)<br>Wzx	CDP–paratose 2–epimerase [*Yersinia mollaretii*]<br>Length: 338<br>84/92(338) 1–338	WP_049608459.1
PL78_00715 (333)<br>WbcC	hypothetical protein [*Yersinia nurmii*]<br>Length: 336<br>77/90(333) 4–336	WP_049598623.1
	putative glycosyltransferase [*Yersinia pseudotuberculosis*]<br>Length: 339<br>59/75(331) 4–334	embCNK90709.1
PL78_00720 (417)<br>Wzy<br>O antigen	histidine kinase [*Yersinia pseudotuberculosis*]<br>Length: 406<br>51/70(404) 1–404	WP_050103368.1
PL78_00725 (337)<br>WbyK<br>RfaB	mannosyltransferase [*Yersinia nurmii*]<br>Length: 337<br>Range 1: 1 to 337<br>0/337(0%)	WP_049598627.1
	mannosyltransferase [*Yersinia pseudotuberculosis*]<br>Length: 337<br>78/88(337)1–337	WP_012304490.1
PL78_00730 (373)<br>Gmd	GDP–mannose 4,6–dehydratase [*Yersinia nurmii*]<br>Length: 373<br>100/100(373) 1–373	WP_049598628.1
	GDP–mannose 4,6–dehydratase [*Yersinia pseudotuberculosis*]<br>Length: 373<br>96/98(373) 1–373	P_011191888.1

PL78_00735 (321)<br>Fcl	GDP–L–fucose synthetase [*Yersinia nurmii*]<br>Length: 323<br>98/99(321) 3–323	embCNE67246.1
	GDP–fucose synthetase [*Yersinia similis*]<br>Length: 321<br>90/94(321) 1–321	WP_054878416.1
PL78_00740 (154)<br>Gmm	GDP–mannose mannosyl hydrolase [*Yersinia nurmii*]<br>Length: 154<br>97/97(154) 1–154	WP_049598631.1
	GDP–mannose mannosyl hydrolase [*Photorhabdus temperata*]<br>Length: 154<br>65/81(154) 1–154	WP_023043826.1
PL78_00745 (468)<br>ManC	mannose–1–phosphate guanyltransferase [*Yersinia nurmii*]<br>Length: 468<br>99/98(468) 1–468	WP_049598633.1
	mannose–1–phosphate guanyltransferase [*Yersinia enterocolitica*]<br>Length: 468<br>87/94(468)1–468	WP_050142517.1
PL78_00750 (247)<br>WbyL	glycosyl transferase [*Yersinia nurmii*]<br>Length: 247<br>98/98(247) 1–247	WP_049598721.1
	glycosyl transferase [*Yersinia mollaretii*]<br>Length: 247<br>76/88(247) 1–247	WP_004873391.1
PL78_00755 (256)<br>ManB	phosphomannomutase [*Yersinia nurmii*]<br>Length: 456<br>99/99(455)1–456	WP_049598635.1
	phosphomannomutase [*Yersinia pseudotuberculosis*]<br>Length: 457<br>85/91(455) 1–455	WP_050093032.1

PL78_00760 (384)<br>Wzz	chain–length determining protein [*Yersinia nurmii*]<br>Length: 384<br>96/98(384)1–384	WP_049598637.1
	chain–length determining protein [*Yersinia ruckeri*]<br>Length: 384<br>76/90(382)1–382	WP_038275531.1
PL78_00765 (436)	inosine/guanosine kinase [*Yersinia nurmii*]<br>Length: 436<br>99/99(436)1–436	WP_049598639.1
	inosine/guanosine kinase [*Yersinia ruckeri*]<br>ength: 436<br>96/98(436)1–436	WP_038243753.1
PL78_00770 (563)	cation:proton antiport protein [*Yersinia nurmii*]<br>Length: 563<br>99/100(563)1–563	WP_049598641.1
	cation:proton antiport protein [*Yersinia intermedia*]<br>Length: 563<br>94/97 (563) 1 to 563	WP_042569300.1
PL78_00775 (404)	fosmidomycin resistance protein [*Yersinia nurmii*]<br>Sequence ID: Length: 404<br>98/98(404)1–404	WP_049598644.1
	fosmidomycin resistance protein [*Yersinia enterocolitica*]<br>Length: 410<br>86/91(410)1–410	WP_050155900.1
PL78_00780 (550)<br>UshA	bifunctional UDP–sugar hydrolase/5'–nucleotidase [*Yersinia nurmii*]<br>Length: 550<br>99/99(550)1–550	WP_049598646.1
	bifunctional UDP–sugar hydrolase/5'–nucleotidase [*Yersinia ruckeri*]<br>Length: 550<br>97/98(550) 1–550	WP_038275532.1

Table S6. *Yersinia entomophaga* countermeasures: ColicinV, Sod, and non–ribosomal peptide synthetase.

Yersinia entomophaga Colicin V.

Locus (Amino Acid Residues)	Closest BlastP Orthologue [Species]<br>Length: Amino Acid Length of Orthologue<br>% Identity/% Similarity (over the Indicated Range of Amino Acid Residues) in Relation to Target Sequence.<br>Protein Domain	Accession Number
PL78_16520 (246)<br>Colicin V	cell division protein DedD [*Yersinia nurmii*]<br>Length: 246<br>95/96(245)1–246	WP_049597003.1
	cell division protein DedD [*Yersinia ruckeri*]<br>Length: 241<br>80/86(247)1–241	WP_038244265.1
PL78_16525 (169)	colicin V production protein [*Yersinia nurmii*]<br>Length: 169<br>99/100(169)1–169	WP_049597004.1
	colicin V production protein [*Yersinia frederikseniir*]<br>Length: 169<br>98/99(169)1–169	WP_050106079.1
PL78_16530 (486)	amidophosphoribosyltransferase [*Yersinia nurmii*]<br>Length: 505<br>99/99(505)1–505	WP_049597005.1
	amidophosphoribosyltransferase [*Yersinia ruckeri*]<br>Length: 505<br>98/99(505)1–505	WP_004717879.1

Yersinia entomophaga superoxide dismutase.

**Locus (Amino Acid Residues)**	**Closest BlastP Orthologue [Species]**<br>**Length: Amino Acid Length of Orthologue**<br>**% Identity/% Similarity (over the Indicated Range of Amino Acid Residues) in Relation to Target Sequence.**<br>**Protein Domain**	**Accession Number**
PL78_05910 (192)<br>SodB	superoxide dismutase [*Yersinia nurmii*]<br>Length: 192<br>99/100(192)1–192	WP_049600140.1
	superoxide dismutase [*Yersinia ruckeri*]<br>Length: 192<br>91/96(192) 1–192	WP_004717636.1
PL78_12235 (208)<br>SodA	superoxide dismutase [*Yersinia nurmii*]<br>Length: 208<br>99/100(208)1–208	WP_049597374.1
	Superoxide dismutase [Mn] [*Yersinia ruckeri* ATCC 29473]<br>Length: 210<br>96/98(208)3–210	gb|EEQ00149.1
PL78_14360 (174)<br>Sod	superoxide dismutase [*Yersinia nurmii*]<br>Length: 174<br>95/95(174) 1–174	WP_049601253.1
	superoxide dismutase [*Yersinia enterocolitica*]<br>Length: 174<br>81/89(174)1–174	WP_046051564.1

Yersinia entomophaga non–ribosomal peptide synthetase.

Locus (Amino Acid Residues)	Closest BlastP Orthologue [Species]<br>Length: Amino Acid Length of Orthologue<br>% Identity/% Similarity (over the Indicated Range of Amino Acid Residues) in Relation to Target Sequence.<br>Protein Domain	Accession Number
PL78_15600 (350)	hydroxymethylpyrimidine/phosphomethylpyrimidine kinase [*Yersinia nurmii*]<br>Length: 266<br>97/98(266) 1–266	WP_049597164.1
	hydroxymethylpyrimidine/phosphomethylpyrimidine kinase [*Yersinia ruckeri*]<br>Length: 266<br>88/93(288) 1–266	WP_038241377.1
PL78_15605 (288)	hypothetical protein [*Yersinia nurmii*]<br>Length: 288<br>95/96(288) 1–288	WP_049596853.1
	hypothetical protein [*Yersinia ruckeri*]<br>Length: 288<br>80/88(288) 1–288	WP_004717978.1
PL78_15610 (152)<br>Ivy	hypothetical protein [*Yersinia nurmii*]<br>Length: 152<br>95/96(152) 1–152	WP_049596854.1
	Inhibitor of vertebrate lysozyme [*Yersinia ruckeri* ATCC 29473]<br>Length: 153 84/92(152) 1–153	gb|EEQ00208.1
PL78_15615 (3939)<br>non–ribosomal peptide synthetase	non–ribosomal peptide synthetase [*Yersinia nurmii*]<br>Length: 3941<br>94/96(3944) 1–3941	WP_049596855.1
	hypothetical protein [*Serratia marcescens*]<br>Length: 4849<br>40/57(3581) 2–3497	WP_060448349.1

PL78_15620 (189)	transposon DNA–invertase [*Yersinia nurmii*]<br>Length: 189<br>99/99(189) 1–189	WP_049596856.1
	DNA invertase [Methylophaga aminisulfidivorans]<br>Length: 191<br>74/87(187)1–187	WP_007143928.1
	DNA invertase [Methylophaga aminisulfidivorans]<br>Length: 191<br>74/87(187)1–187	WP_007143928.1

Yersinia entomophaga non–ribosomal peptide synthetase.

**Locus (Amino Acid Residues)**	**Closest BlastP Orthologue [Species]**<br>**Length: Amino Acid Length of Orthologue**<br>**% Identity/% Similarity (over the Indicated Range of Amino Acid Residues) in Relation to Target Sequence.**<br>**Protein Domain**	**Accession Number**
PL78_04160 (191)	HutD family protein [*Yersinia nurmii*]<br>Length: 191<br>88/93(191)1–191	WP_049597639.1
	HutD family protein [*Yersinia ruckeri*]<br>Length: 190<br>72/82(187) 1–186	WP_042524807.1
PL78_04165 (1844)<br>non–ribosomal peptide synthetase	non–ribosomal peptide synthetase [*Yersinia nurmii*]<br>Length: 1844<br>92/95(1844)1–1844	WP_049597640.1
	pyochelin synthetase [Brevibacillus laterosporus]<br>Length: 1838<br>47/65(1838)8–1837	WP_031412270.1

PL78_04170 (78)	MULTISPECIES: transposase [*Enterobacteriaceae*]<br>Length: 92<br>97/98(92)1–92	WP_012442396.1
	transposase [*Yersinia ruckeri*]<br>Length: 92<br>95/96(92)1–92	WP_038245923.1
PL78_04175 (283)	transposase [*Yersinia pekkanenii*]<br>Length: 283<br>99/99(283) 1–283	WP_049615490.1
	integrase [*Erwinia tasmaniensis*]<br>Length: 283<br>99/99(283) 1–283	WP_012442397.1
